# Auxiliary-directed etherification of sp^2^ C–H bonds under heterogeneous metal–organic framework catalysis: synthesis of ethenzamide†

**DOI:** 10.1039/c7ra12010a

**Published:** 2018-01-16

**Authors:** Chau B. Tran, Xuan N. T. Duong, Huy D. Lu, Thu T. V. Cao, Thanh Truong

**Affiliations:** Department of Chemical Engineering, Ho Chi Minh University of Technology, VNU-HCM 268 Ly Thuong Kiet, District 10 Ho Chi Minh City Viet Nam truongvuthanh@gmail.com tvthanh@hcmut.edu.vn +84 8 38637504 +84 8 38647256 ext. 5681

## Abstract

An efficient protocol for 8-aminoquinoline assisted alkoxylation and phenoxylation of sp^2^ C–H bonds under heterogeneous catalysis was developed. The optimal conditions employed Cu-MOF-74 (20%), K_2_CO_3_ base, pyridine ligand or dimethyl formamide solvent, and O_2_ oxidant at 80 °C or 100 °C for 24 hours. Cu-MOF-74 revealed remarkably higher activity when compared with other previously commonly used Cu-MOFs in cross coupling reactions, supported copper catalysts, and homogeneous copper salts. The reaction scope with respect to coupling partners included a wide range of various substrates. Interestingly, the developed conditions are applicable for the synthesis of high-profile relevant biological agents from easily accessible starting materials. Furthermore, a leaching test confirmed the reaction heterogeneity and the catalyst was reused and recycled at least 8 times with trivial degradation in activity.

## Abstract 2

An efficient protocol for 8-aminoquinoline assisted alkoxylation and phenoxylation of sp^2^ C–H bonds under heterogeneous catalysis was developed.

## Graphical abstract

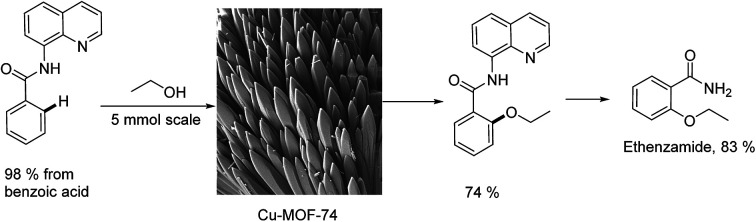

## 1. Introduction

Aryl ethers have been frequently found in natural products, agricultural chemicals, pharmaceuticals, and biologically active compounds.^1–3^ Common methodologies to access these C–O bonds involved the use of reactions under transition metal catalysis such as Buchwald–Hartwig or Chan–Evans–Lam coupling.^4–6^ However, the utilization of pre-functionalized starting materials such as aryl halides or aryl boronic acids limited their applications. Transition-metal-catalyzed C–H etherification has recently been recognized as the most direct way and has attracted considerable attention.^7^ In the early stages, most studies of direct etherification of sp^2^ C–H bonds containing directing groups have been performed using palladium or ruthenium catalysts.^8,9^ Lately, first-row transition metals have become the target of extensive research due to their abundance and low toxicity. In particular, an elegant work by Yu demonstrated the etherification/hydroxylation of 2-phenylpyridine by employing Cu(ii) salts and oxygen oxidant.^10^ The approaches to aryl ethers *via* copper-catalyzed *ortho*-alkoxylation of benzoic acid derivatives and 2-arylpyridine were subsequently discovered.^11,12^ However, stoichiometric amount of silver salts were required as oxidants and the directing groups were not removable. Recently, though report utilized the (pyridin-2-yl)isopropyl amine (PIP) directing group with excellent functionality tolerance was disclosed, the protocol was only applicable for methoxylation of arenes and heteroarenes.^13^ A general method for alkoxylation and phenoxylation of β-sp^2^ C–H bonds of benzoic acid derivatives and γ-sp^2^ C–H bonds of amine derivatives were developed by Daugulis group.^14^ To the best of our knowledge, the heterogeneous catalysts for these useful reactions still remain unexplored. Therefore, the development of more practical methods avoiding the contaminated metals from final products is highly demanding, especially in pharmaceutical industry.

The tunable and versatile combinations of metal ions or clusters with polyfunctional organic linkers allow metal–organic frameworks (MOFs) to acquire applications in a variety of ways.^15–18^ The last few years have witnessed the substantial amount of chemical research focusing on the catalytic activity of MOFs toward modern organic transformations.^19–22^ Though possessing high surface area and high porosity, challenges of using MOFs catalysts included the inclusion of large organic molecules through the pore aperture. Thus, the development of series of metal–organic frameworks, MOF-74, possessing large pore apertures opened up great opportunities for catalysis application, especially for complex organic compounds.^23^ Our group has demonstrated the activity of Cu-MOF-74 for the amidation by amine and phenylglyoxal.^24^ Taking into the next level by employing more complex structures and reactions, we herein disclose a general route for auxiliary-directed etherification of sp^2^ C–H bonds using Cu-MOF-74 as an efficient heterogeneous catalyst. Besides the use of recyclable material, the methodology is applicable for aliphatic alcohol and phenol derivatives. Interestingly, the synthesis of targeted bioactive compounds was also performed for the first time under heterogeneous conditions.

## 2. Experimental

### 2.1. Synthesis of Cu-MOF-74

The optimization for Cu-MOF-74 synthesis was placed in ESI (Table S1†). In a typical preparation, 2,5-dihydroxyterephthalic acid (H_2_dhtp) (0.186 g, 0.97 mmol) and copper nitrate trihydrate (CuNO_3_·3H_2_O) (0.5 g, 2.07 mmol) were dissolved separately in a mixture of *N*,*N*-dimethylformamide (DMF) and 2-propanol (20 : 1). The latter, blue solution containing copper salt, was added slowly into the stirring ligand solution for approximately 30 minutes. The resulting solution was then distributed to 8 mL vials, and placed in an isothermal oven at 85 °C for 18 hours. The sample was cooled down to room temperature and the solid product was removed by decanting with mother liquor. The reddish needle-shaped crystals were washed with DMF (3 × 20 mL) for three days and solvent exchanged with methanol (3 × 20 mL) over a three-day period. The material was then evacuated under vacuum at 150 °C in 8 hours, Cu-MOF-74 (0.269 g, 62% based on H_2_dhtp) was obtained in the form of reddish black crystals.

### 2.2. Catalytic studies

Reported values in manuscript are average numbers of at least 2 runs and the error was less than 7%. In a typical experiment, a mixture of *N*-(quinolin-8-yl)benzamide (0.2 mmol, 1.0 equiv.) and K_2_CO_3_ (0.2 mmol, 1.0 equiv.) was added into a 8 mL screwed tube with the pre-determining amount of Cu-MOF-74 catalyst (0.04 mmol, 20 mol%). Alcohol (1.6 mL) and pyridine (0.40 mL) was added alternately, and the mixture was stirred at 80 °C for 24 hours under oxygen atmosphere. When the reaction completed, diphenyl ether (0.2 mmol) was added into the reaction mixture as an internal standard for GC yield calculation. The organic components were then extracted into water (1.0 mL) and ethyl acetate (3.0 mL). The organic phase was dried by anhydrous sodium sulfate and was characterized by GC analysis with reference diphenyl ether. The remaining organic portion concentrated under reduced pressure and the residue was purified by flask chromatography. Finally, products were further characterized by ^1^H NMR, ^13^C NMR.

For phenoxylation: K_2_CO_3_ (0.4 mmol, 2 equiv.), and Ar–OH (0.4 mmol, 2 equiv.) were employed in DMF solvent (2.0 mL) at 100 °C.

## 3. Results and discussion

**Fig. 1:**
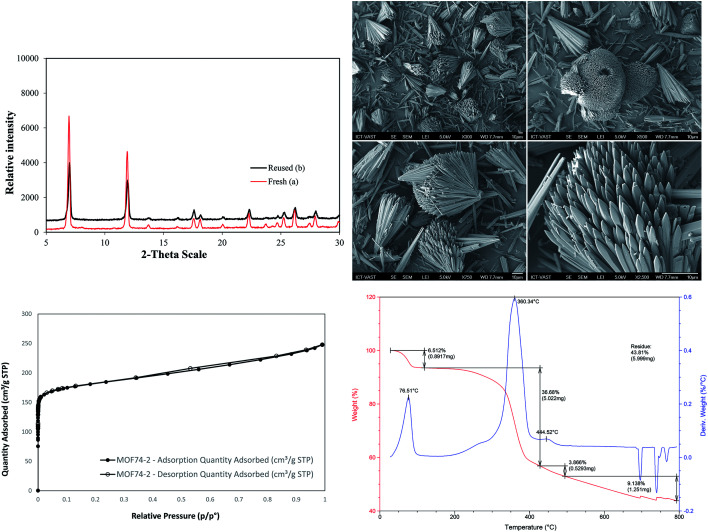
PXRD, SEM micrograph, adsorption/desorption isotherm, and TGA of synthesized Cu-MOF-74.

The Cu-MOF-74 was successfully synthesized with 62% yield by a solvothermal method according to literature procedure.^24,25^ The characterization was elucidated by different techniques including powder X-ray diffraction pattern (PXRD), scanning electron microscopy (SEM), thermal gravimetric analysis (TGA), inductively coupled plasma optical emission spectrometry (ICP-OES), and nitrogen physisorption measurements (Fig. 1 and S1†). The analysis results matched well with those obtained in previous reports.^25^ In particular, PXRD is in good agreement with simulated pattern showing the typical reflections of MOF-74 phase with open metal sites. The adsorption/desorption isotherm supported the permanent micro-porosity with Langmuir surface area of 1289 m^2^ g^−1^, a pore volume of 0.39 cm^3^ g^−1^ and an adsorption average pore size of about 11.2 Å. SEM analysis showed the homogeneity of paddle wheel structure. TGA of activated Cu-MOF-74 shows high thermal stability (>300 °C) and the measured mass percent of residue CuO is consistent with the EA data. ICP-OES provided 27.5% copper content which is close to the calculated value of 28.2%.

In optimization studies, reactions of *N*-benzoyl-8-aminoquinoline with ethanol were conducted (Table 1). Mimicking reported conditions of homogeneous copper salt catalyst did not lead to the formation of desired product (entry 1). The utilization of inorganic bases provided better efficiency and up to 49% yield was obtained in K_2_CO_3_ (entries 2–5). Specifically, decomposition of starting materials was observed in strong organic bases (entries 2, 3). Inorganic bases that possessing lower p*K*_a_ than K_2_CO_3_ such as Li_2_CO_3_ and CH_3_COOK were not sufficient (entries 5, 6) while stronger bases than K_2_CO_3_ led to the formation of di-alkoxylated products as well as partially deconstruction of starting materials (entries 7–9). Interestingly, no products were detected in tested co-solvents with different polarity in the absence of pyridine (entries 10–12). It is hypothesized that pyridine facilitated the deprotonation of alcohol as base and reductive elimination by decreasing the cone angle.^26^ Increasing reaction temperature provided similar yield while significant drop was observed when reaction was run at 60 °C (entries 13, 14). Though slightly higher yield was achieved with K_2_S_2_O_8_, deconstruction of MOFs was revealed by measuring the concentration of copper in reaction filtrate in conjunction with decomposition of starting material (entry 16). With regard to oxidant, best results were provided in oxygen atmosphere (entries 15, 17). Using more catalyst offered trivial impact (entry 18). Gratifyingly, 80% yield of product was generated when the reaction was carried out at 0.5 M concentration (entry 21). High reaction selectivity was expected due to the insignificant differences between reaction conversion and yield. Using more or less than 1 equivalent of K_2_CO_3_ offered lower yields and (entries 23–24). With regard to reaction kinetics, 24 hours were required to reach optimal results in most tested parameters (entry 25 and Fig. S5†).

**Table 1:** Optimization of reaction conditions^a^

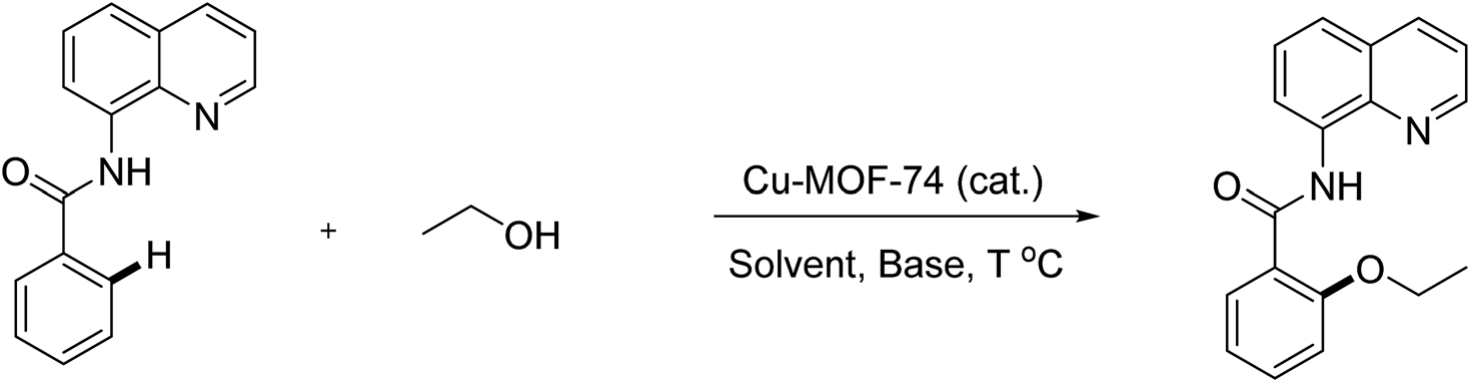							
Entry	Base	Ligand, volume (mL)	Temp. (°C)	Oxidant	Catalyst amount (mol%)	Conversion (%)	Yield^b^ (%)
1	Tetramethyl guanidine	Pyridine, 1	80	O_2_/air	20	<5	<5
2	^*t*^BuOLi	Pyridine, 1	80	O_2_	20	35	<5
3	^*t*^BuOK	Pyridine, 1	80	O_2_	20	69	<5
4	K_2_CO_3_	Pyridine, 1	80	O_2_	20	50	49
5	Li_2_CO_3_	Pyridine, 1	80	O_2_	20	44	42
6	CH_3_COOK	Pyridine, 1	80	O_2_	20	17	15
7	Cs_2_CO_3_	Pyridine, 1	80	O_2_	20	28	18
8	KHCO_3_	Pyridine, 1	80	O_2_	20	37	14
9	K_3_PO_4_	Pyridine, 1	80	O_2_	20	52	21
10	K_2_CO_3_	Toluene, 1	80	O_2_	20	<5	<5
11	K_2_CO_3_	Dioxane, 1	80	O_2_	20	<5	<5
12	K_2_CO_3_	DMF, 1	80	O_2_	20	<5	<5
13	K_2_CO_3_	Pyridine, 1	100	O_2_	20	55	50
14	K_2_CO_3_	Pyridine, 1	60	O_2_	20	26	24
15	K_2_CO_3_	Pyridine, 1	80	Air	20	33	29
16^c^	K_2_CO_3_	Pyridine, 1	80	K_2_S_2_O_8_	20	95	53
17	K_2_CO_3_	Pyridine, 1	80	TBHP	20	25	15
18	K_2_CO_3_	Pyridine, 1	80	O_2_	15	34	31
19	K_2_CO_3_	Pyridine, 1	80	O_2_	25	52	50
20	K_2_CO_3_	Pyridine, 0.6	80	O_2_	20	67	62
**21**	**K**_**2**_ **CO**_**3**_	**Pyridine, 0.4**	**80**	**O**_**2**_	**20**	**87**	**80**
22	K_2_CO_3_	Pyridine, 0.2	80	O_2_	20	58	54
23^d^	K_2_CO_3_	Pyridine, 0.4	80	O_2_	20	68	63
24^e^	K_2_CO_3_	Pyridine, 0.4	80	O_2_	20	58	55
25^f^	K_2_CO_3_	Pyridine, 0.4	80	O_2_	20	52	48

a EtOH (20 equiv.), 0.2 mmol scale, base (1 equiv.), 24 h.

b GC yields.

c Leaching of copper and decomposition of starting material were observed.

d 1.5 equiv. of base.

e 0.5 equiv. of base.

f Reaction in 12 h. Kinetic studies of entries and more other information were placed in ESI.

To emphasize the catalytic activity of Cu-MOF-74, we decided to test several open metal-site Cu-MOFs with different aperture pore for the oxidative etherification (Table 2). In general, high reaction selectivity was observed in all cases. Surprisingly, other previously commonly used Cu-MOFs in cross coupling reactions failed to provide sufficient amount of products (entries 2–6).^27–29^ This could be rationalized by the exceptional large aperture pore size of Cu-MOF-74, especially when starting materials possess large molecular size. Magnetic nanoparticle CuFe_2_O_4_ as well as traditional Cu/zeolite or Cu/ZSM-5 were showed to be inefficient (entries 7–9). No detected amount of products with MOF-74 incorporated other metals such as Co, Ni, and Zn indicated the necessity of copper site (entries 10–12). Notably, CuBr_2_ generated best results with 73% yields while <35% yields were obtained with other tested bare copper salts such as Cu(OAc)_2_, Cu(acac)_2_, CuI, and Cu(OBz)_2_ (entries 13–17). It is worth mentioning that the utilization of heterogeneous catalytic system, especially in industry, is preferred due to the ease in separation, recyclability, and avoiding the removal of contaminated metal in final products.^30,31^

**Table 2:** Reactions with other catalysts^a^

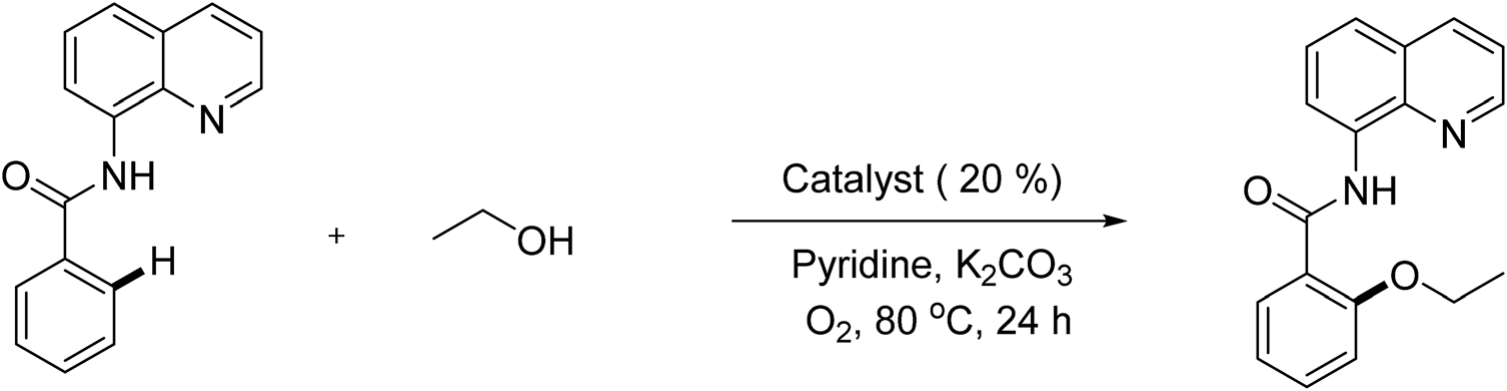					
Entry	Type	Catalyst	Coordination number	Conversion (%)	GC yield (%)
1	Heterogeneous Cu-based catalysts	Cu-MOF-74	6 (ref. 32)	88	80
2		Cu(BDC)	4 (ref. 33)	26	20
3		Cu_2_(BDC)_2_ (DABCO)	5 (ref. 34)	25	24
4		Cu_2_(BDC)_2_(BPY)	5 (ref. 35)	21	15
5		Cu_2_(BPDC)_2_(BPY)	5 (ref. 36)	19	17
6		Cu_2_(OBA)_2_(BPY)	5 (ref. 37)	17	13
7		CuFe_2_O_4_		<5	<5
8		Cu/zeolite X		34	31
9		Cu/ZSM-5		21	17
11	Other MOFs	MOF-74-Ni		<5	<5
11		MOF-74-Co		<5	<5
12		MOF-74-Zn		<5	<5
13	Homogeneous Cu(ii) salts	Cu(OAc)_2_		16	11
14		CuBr_2_		88	73
15		Cu(acac)_2_		34	19
16		CuI		41	34
17		Cu(OBz)_2_		33	28

a Volume of ligand 0.4 mL, 0.2 mmol scale, K_2_CO_3_ (1 equiv.).

With respect to catalyst structure analysis, Cu-MOF-74 possesses 6 coordination numbers, which is substantially high as compared to other common Cu-MOFs with paddle wheel units and owning open metal site (Table 2).^32–37^ Polynuclear copper complexes with paddle-wheel units were reported to favorably exist in octahedral geometry.^38,39^ In particular, six coordination sites of Cu-MOF-74 include solvent (01 coordination), Cu–Cu bridge (01 coordination), and carboxylate linkers (04 coordination). Coordinating with solvent is reversible and the carboxylate can chelate with copper by 01 coordination site. It was reported that coordination number of copper(ii) ranged from 02 to 10.^40^ In addition, structure of MOFs could remain intact upon cooling though coordination of MOFs during reaction progress could be changed.^41^ Therefore, the Cu site is expected to have vacancies for association/dissociation with additional ligand, which is in good agreement with disclosed mechanism about chelation of copper with directing group to activate the *ortho* C–H bond.^42,43^ Furthermore, good activity of Cu-MOF-74 could be rationalized by following factors: (1) high coordination number of metal site favors the reductive elimination step,^26^ (2) additional coordination of –OH group from linker in Cu center facilitate the C–H activation by introducing the electron rich environment.^44^

Reaction scope with respect to coupling partners was performed to investigate the generality of optimized conditions (Table 3). Pyridine was employed as ligand for all alkoxylation reactions. For phenoxylation, our optimization screening pointed out that DMF solvent was best while 2 equivalents of K_2_CO_3_ were required (Table S2†). Phenols are more acidic than aliphatic alcohol and reductive elimination step of phenoxyl complex is more facile than corresponding alkoxyl ones.^26,44^ Thus, the utilization of pyridine ligand could be avoided. Alkoxylation by ethanol, methanol, and *n*-butanol afforded good yields (entries 1–3). Interestingly, C–H activation by secondary alcohol was possible and product was obtained in acceptable yield (entry 4). The optimized condition was applicable to alcohol bearing double bonds (entry 5). Selective mono-alkoxylation by ethylene glycol gave product in 88% yield (entry 6). For phenoxylation, phenol and its derivatives bearing *ortho*-, *meta*-, or *para*-methyl substituent are reactive (entries 7–10). C–H activation by electron-rich or electron-poor phenols generated C–O products with good functional group tolerance (entries 11–14). The reaction scope with respect to aminoquinoline benzamides is investigated (entries 15–18). In particular, benzamides with various substituents such as *p*-chloro, *p*-methoxy, *o*-methyl, and *m*-bromo are compatible and phenoxylated products were obtained in reasonable yields.

**Table 3:** Reaction scope for alkoxylation^a^

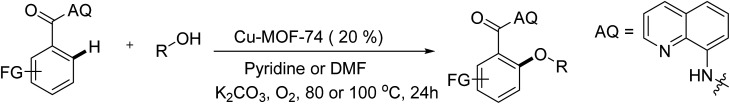					
Entry	Product	Yield (%)	Entry	Product	Yield (%)
1	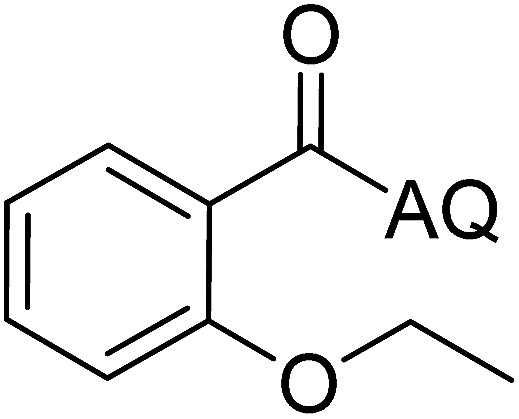	77	10	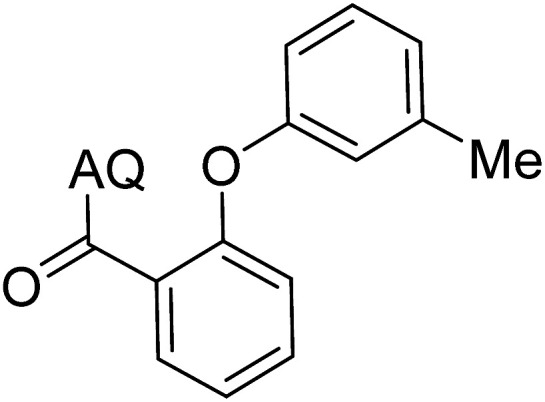	48
2	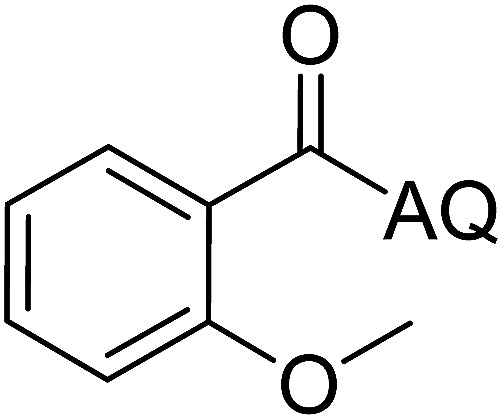	87	11	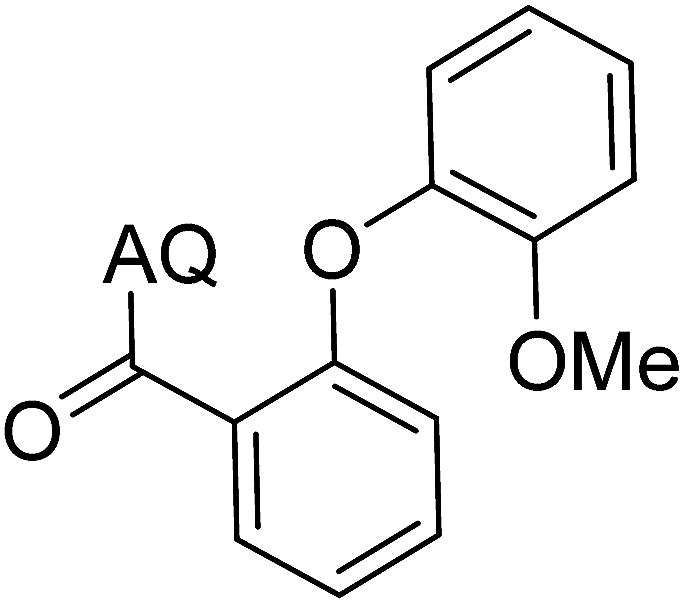	76
3	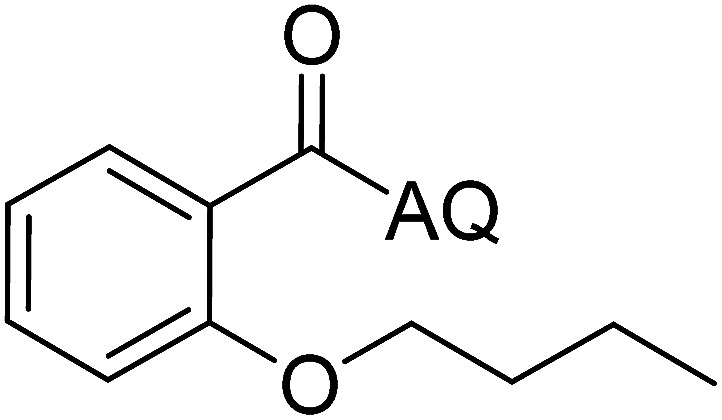	68	12	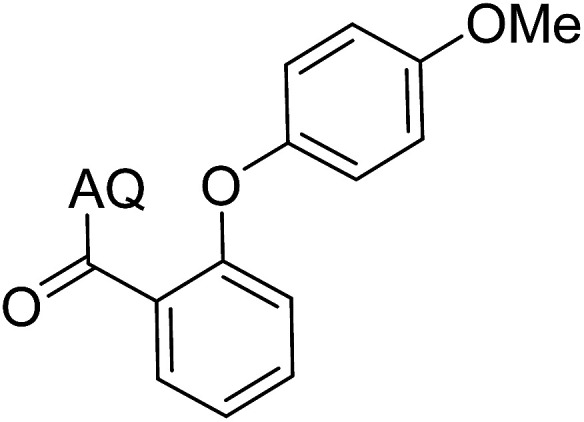	63
4	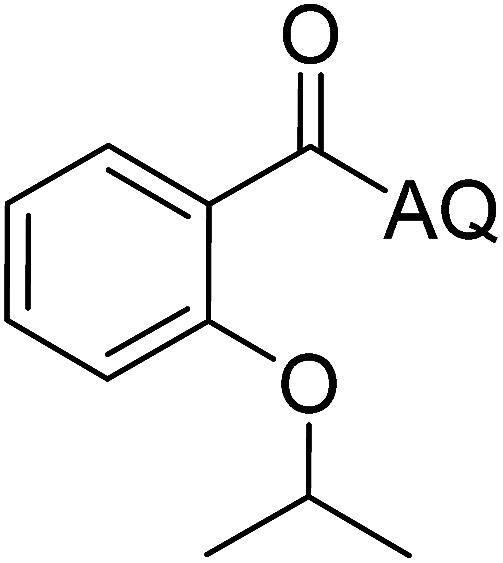	35	13	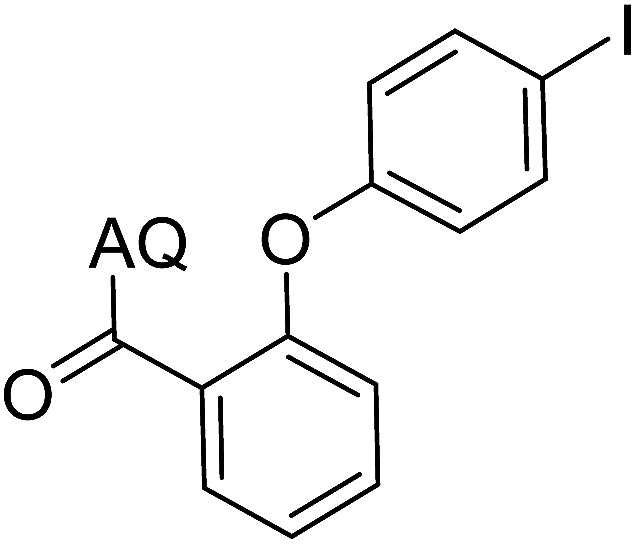	59
5	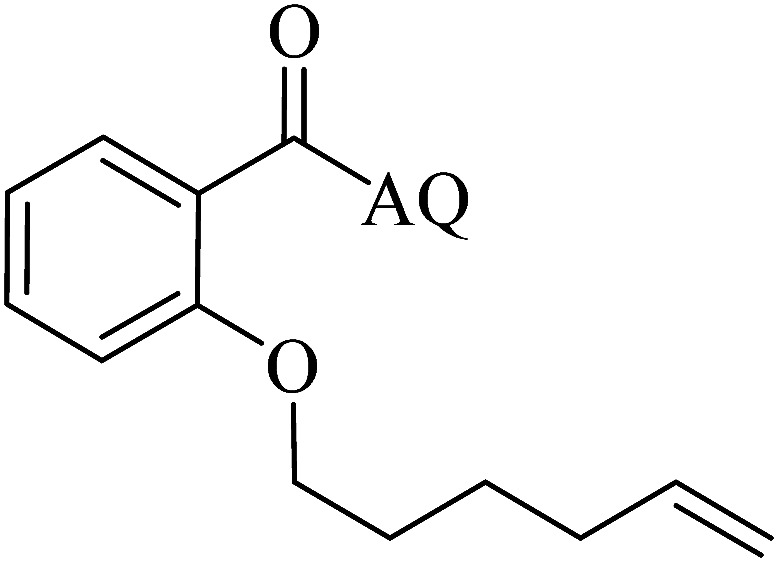	41	14	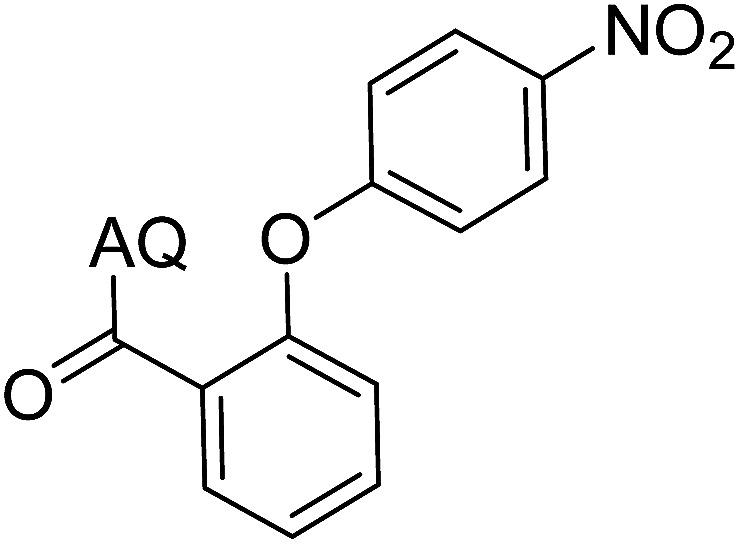	70
6	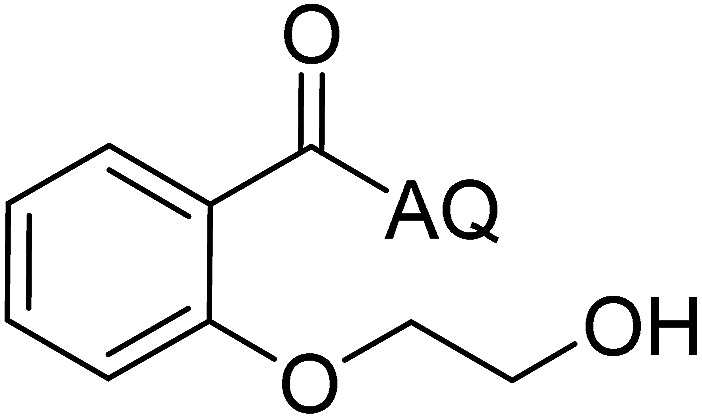	88	15	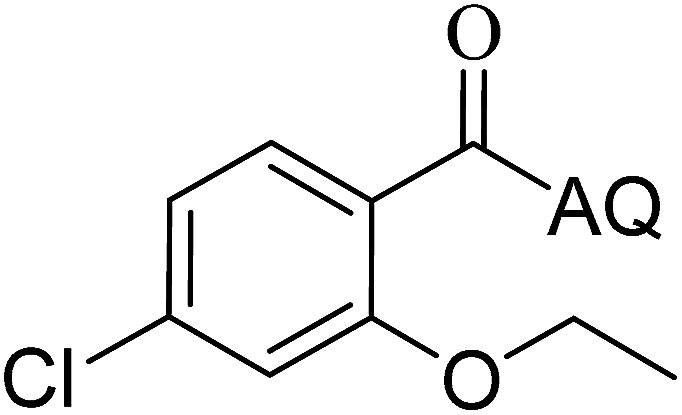	86
7	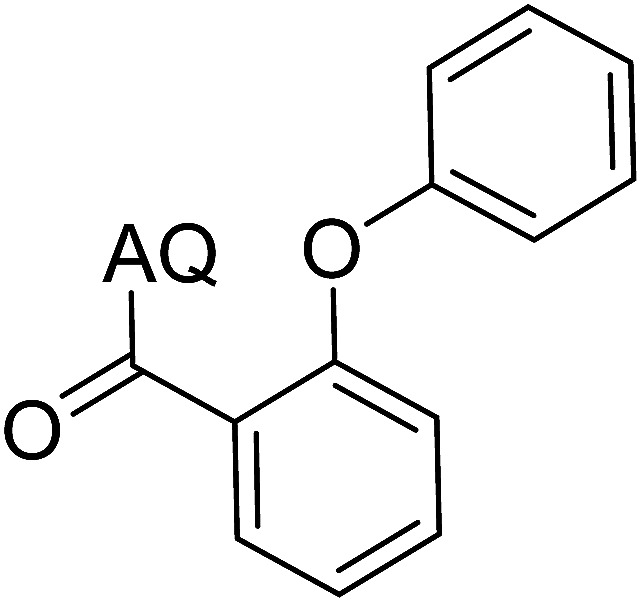	73	16	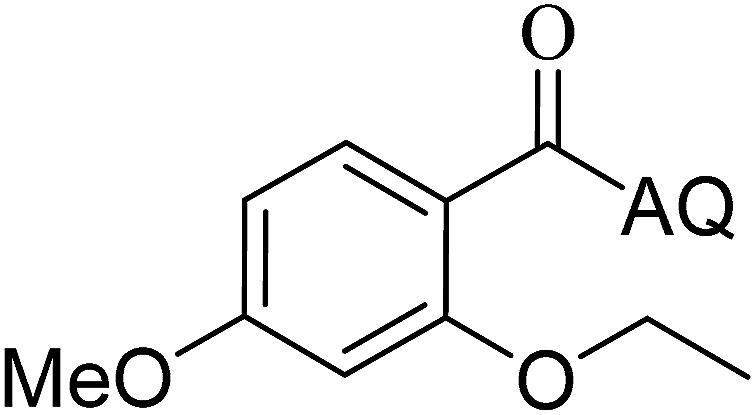	63
8	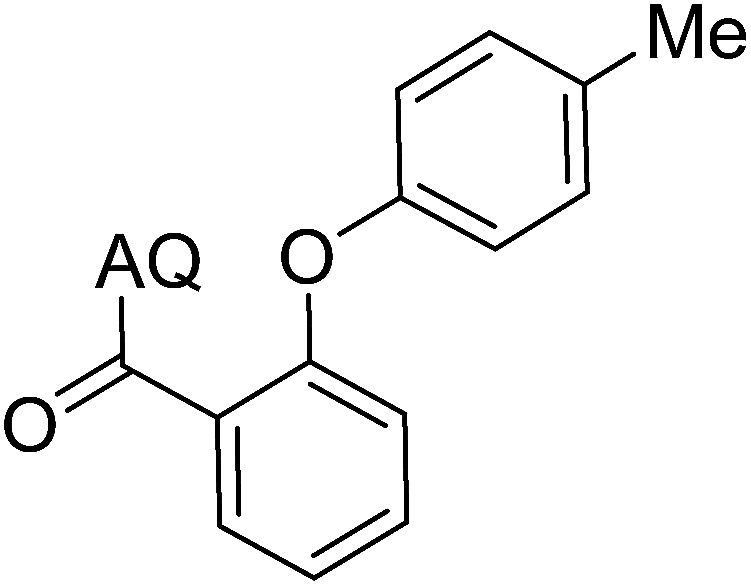	51	17	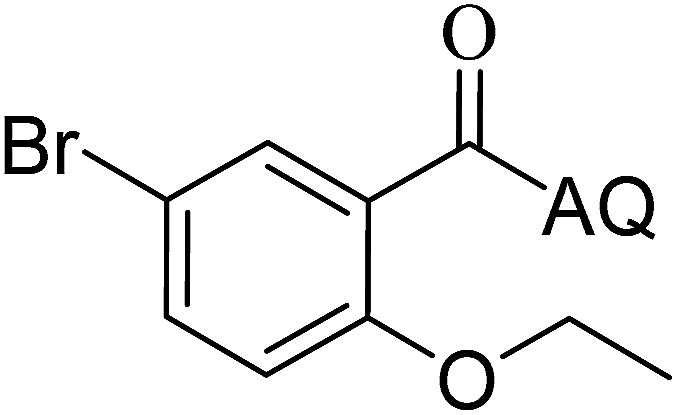	52
9	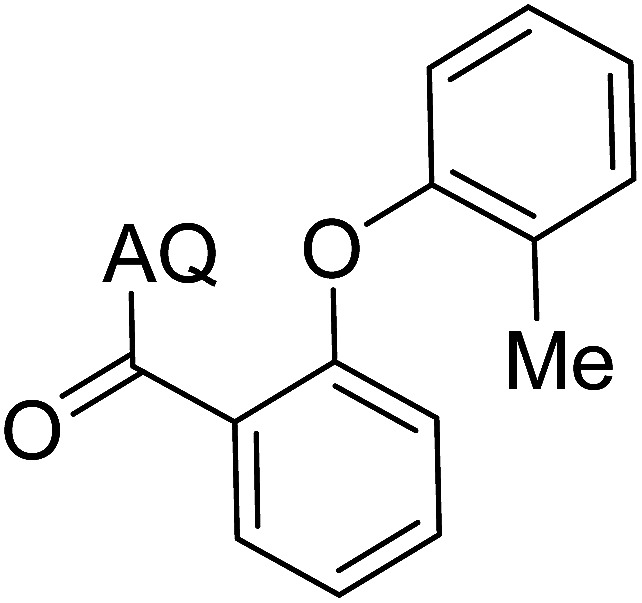	56	18	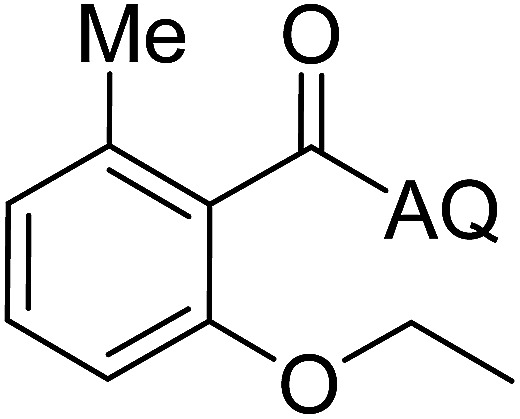	58

a 0.2 mmol scale. For alkoxylation: ROH (20 equiv.), volume of pyridine ligand 0.4 mL, K_2_CO_3_ (1 equiv.), 80 °C; for phenoxylation: Ar–OH (2 equiv.), volume of DMF solvent 2.0 mL, K_2_CO_3_ (2 equiv.), 100 °C.

**Scheme 1:**
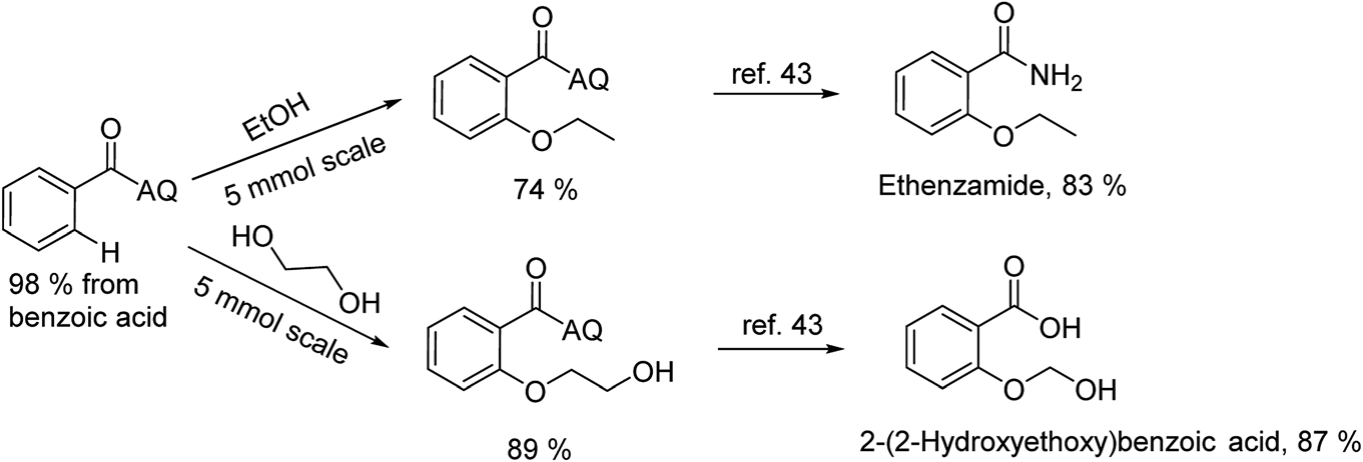
Reactions with large scale and the synthesis of targeted bioactive compounds.

To emphasize the potential of optimized conditions in practical applications, the synthesis of high-profile relevant biological agents was performed (Scheme 1). Gratifyingly, the reactions can be scaled up at least 25-fold with trivial loss of yield. Ethenzamide, an analgesic and anti-inflammatory drug, could be achieved by cleavage of the 8-aminoquinoline directing group in aqueous ammonia.^45^ The procedure provided a novel 3-steps approach of ethenzamide from cheap and readily available benzoic acid with total 60% yield.^46^ In addition, an insulin inhibitor, 2-(2-hydroxyethoxy)benzoic acid, was targeted from benzoic acid and ethylene glycol in 76% yield.^47^ It is noted that these compounds were not previously synthesized using C–H alkoxylation methodology or/and heterogeneous catalysts. The combination of recyclable solid catalyst and avoiding pre-functional starting materials makes the protocol more economically practical.

**Fig. 2:**
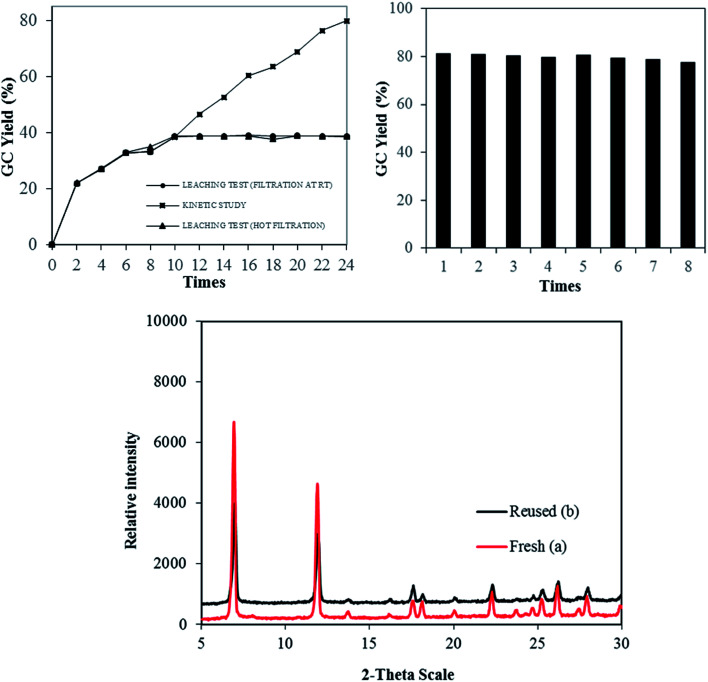
Leaching test and recycling studies.

The stability and heterogeneity of Cu-MOF-74 were then examined. Control experiments were subsequently carried out by repeatedly separating the solid catalyst from the resulting mixture and employed for next runs under identical conditions. No significant further formation of product was detected after catalyst was removed by hot filtration or filtration at room temperature. In conjunction with the concentration of Cu (<1.0 ppm) in reaction filtrate, the results revealed that the catalysis from leached active copper species in the liquid phase is unlikely. Subsequently, the Cu-MOF-74 could be recovered and reused at least 08 consecutive times without a significant degradation in catalytic activity. Specifically, a yield of 78% was still obtained in the 8^th^ run (Fig. 2). Furthermore, the crystallinity of used catalyst, as shown by PXRD, was almost intact as compared to that of fresh catalyst.

## 4. Conclusions

In summary, we have developed a heterogeneous catalytic system for auxiliary-directed alkoxylation and phenoxylation of sp^2^ C–H bonds. The optimized conditions involved the utilization of Cu-MOF-74 catalyst (20%), K_2_CO_3_ base, oxygen as terminal oxidant, pyridine ligand or DMF solvent with 0.5 M concentration at 80 °C or 100 °C in 24 hours. The Cu-MOF-74 exhibited excellent activity among other previously commonly used Cu-MOFs in cross coupling reactions, traditional heterogeneous copper catalyst, and tested bare copper salts. The method shows high generality with wide range of coupling partners and good functional group tolerance. Interestingly, the potential of the reported route in practical applications was demonstrated in the synthesis of bioactive compounds from readily accessible benzoic acids starting materials in acceptable yields. Heterogeneity of Cu-MOF-74 was confirmed by leaching test and recycling studies.

## Conflicts of interest

There are no conflicts to declare.

## Supplementary Material

RA-008-C7RA12010A-s001
