# Supplementary material for: Auxiliary-directed etherification of sp2 C–H bonds under heterogeneous metal–organic framework catalysis: synthesis of ethenzamide
Source: RSC Adv. 2018 Jan 16;8(5):2829–36. doi: 10.1039/c7ra12010a (PMC9077472; doi:10.1039/c7ra12010a)

Electronic Supplementary Material (ESI) for RSC Advances.
This journal is © The Royal Society of Chemistry 2017

Auxiliary-directedetherification of sp^2C-H bonds under heterogeneousmetal-organicframeworkcatalysis: synthesis of Ethenzamide

Chau B. Tran,† Xuan N. T. Duong,† Huy D. Lu, Thu T. V. Cao, Thanh Truong*

Department of Chemical Engineering, Ho Chi Minh University of Technology, VNU-HCM,

268 Ly ThuongKiet, District 10, Ho Chi Minh City, Viet Nam

† These authors contributed equally.

*Email: truongvuthanh@gmail.com or tvthanh@hcmut.edu.vn

SUPPORTING INFORMATION

Materials and instrumentation

All reagents and starting materials were obtained commercially from Sigma-Aldrich and Merck, and were used as received without any further purification unless otherwise noted. Powder X-ray diffraction patterns (PXRD) were collected using a BrukerD8 Advance equipped with a Ni filtered Cu Kα radiation (λ = 1.54178 Å) source. The diffractometer was also equipped with an anti-scattering shield that prevented incident diffuse radiation from hitting the detector. Nitrogen physisorption measurements were conducted using a Micromeritics 2020 volumetric adsorption analyzer system. Samples were pretreated by heating under vacuum at 150 °C for 3 h. A NetzschThermoanalyzerSTA 409 was used for thermogravimetric analysis (TGA) with a heating rate of 10 °C/min under a nitrogen atmosphere. Scanning electron microscopy studies were conducted on a JSM 740 scanning electron microscope (SEM). Transmission electron microscopy studies were performed using a JEOLJEM 1400 Transmission Electron Microscope (TEM) at 100 kV. Fourier-transform infrared (FT-IR) spectra were obtained on a Nicolet 6700 instrument, with samples being dispersed on potassium bromide pallets. The chemisorption experiments were studied in a Micromeritics 2020 analyzer. Gas chromatographic (GC) analyses were performed using a Shimadzu GC 2010-Plus equipped with a flame ionization detector (FID) and an SPB-5 column (length = 30 m, inner diameter = 0.25 mm, and film thickness = 0.25 μm). The temperature program for GC analysis held samples at 120 °C for 1 min; heated them from 120 to 180 °C at 50 °C/min; held them at 180 °C for 1 min; heated them from 180 to 280 °C at 50 °C/min and held them at 280 °C for 3 min. Inlet and detector temperatures were set constant at 280 °C. 1,2-Dichlorobenzene was used as an internal standard to calculate reaction yields. GC-MS analyses were performed using a Hewlett Packard GC-MS 5972 with a RTX-5MS column (length = 30 m, inner diameter = 0.25 mm, and film thickness = 0.5 μm). The

temperature program for GC-MS analysis heated samples from 60 to 280 °C at 10°C/min and held them at 280 °C for 2 min. Inlet temperature was set constant at 280 °C. MS spectra were compared with the spectra gathered in the NIST library.^{1}H and ^{13}CNMR spectra were recorded in $CDCl_3$ using TMS as an internal standard on a BrukerNMR spectrometer at 500 MHz and 125 MHz, respectively.

To investigate the recyclability of Cu-MOF-74, the catalyst was separated from the reaction mixture by simple filtration, washed with copious amounts of DMF and methanol, dried 150°C under vacuum in 4 h.

Leaching test procedure

For the leaching test, a catalytic reaction was stopped after 10 hours. The resulting mixture was analyzed by GC to calculation reaction yield. The mixture was then filtered and to remove the solid catalyst. To provide better accuracy on catalyst heterogeneity, both filtration at room temperature and hot filtration were conducted. The filtrate was then stirred for a further 14 hours at optimized reaction conditions. Reaction progress, if any, was monitored in both cases by GC as previously described.

Table S1. Catalyst synthesis

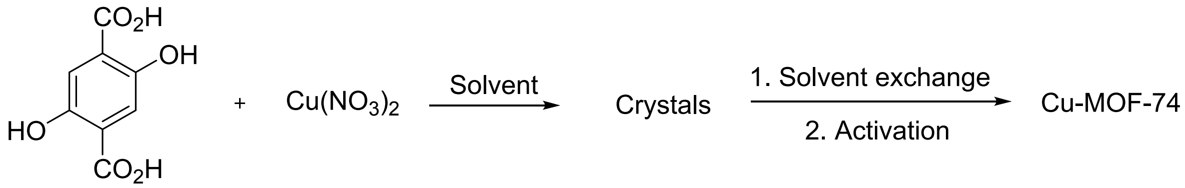

Entry	Reaction solvent	Metal/linker ratio	Exchange solvent	Yield
1	DMF/H_2O	2.1	MeOH	58
**2**	**DMF/Isopropanol**	**2.1**	**MeOH**	**62**
3	DMF/Isopropanol	1.8	MeOH	47
4	DMF/Isopropanol	2.3	MeOH	64
5	DMF/Isopropanol	2.1	Isopropanol	58

Catalysts characterization

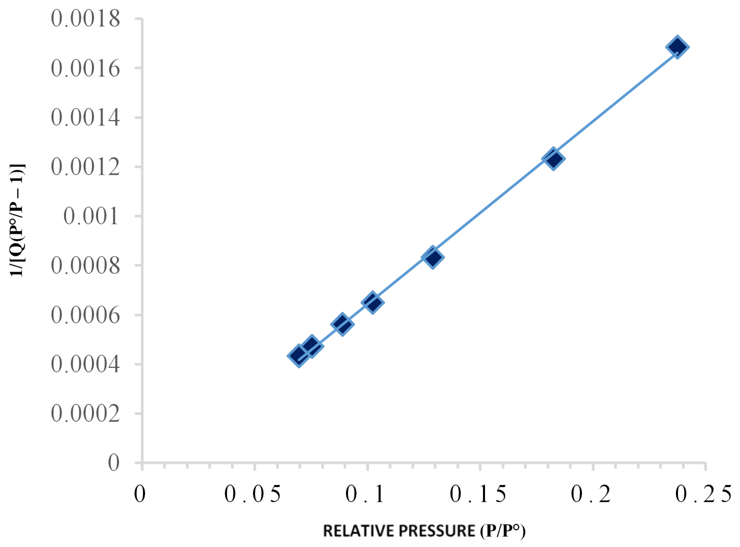

Fig. S1. Nitrogen physisorptionmeasurements

Optimization

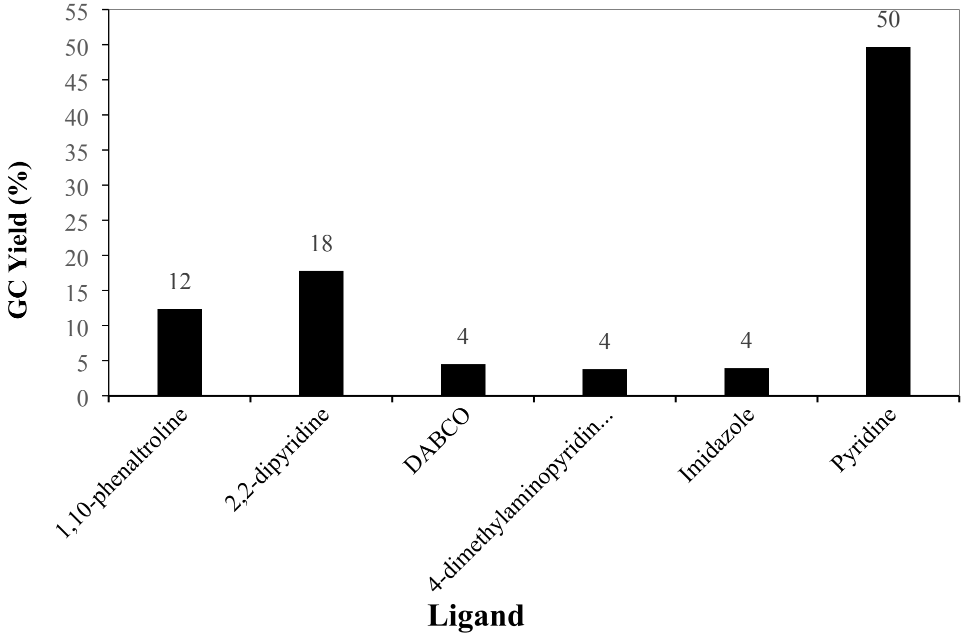

Fig. S2. Effect of ligand on reaction yield

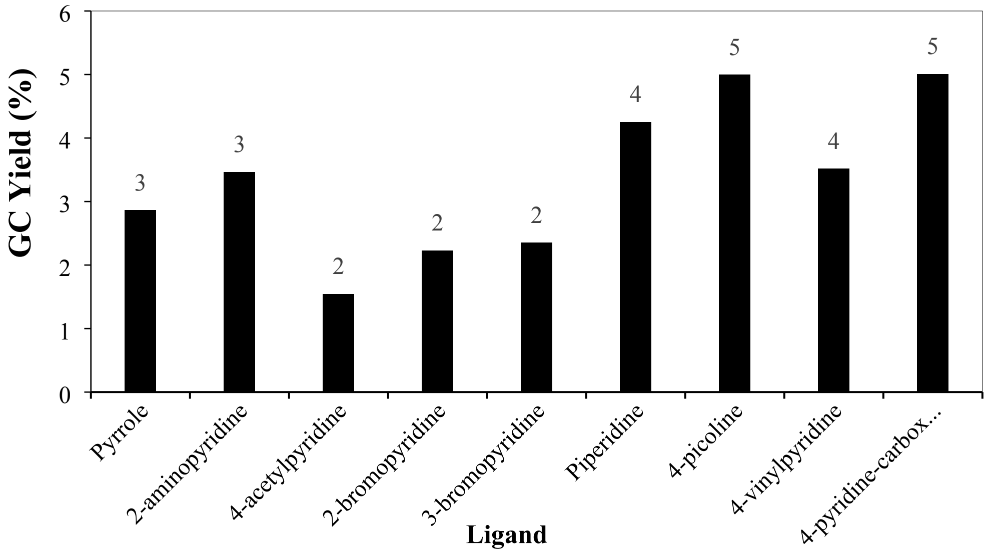

Fig. S3. Effect of mono-pyridines derivetives as ligand on reaction yield

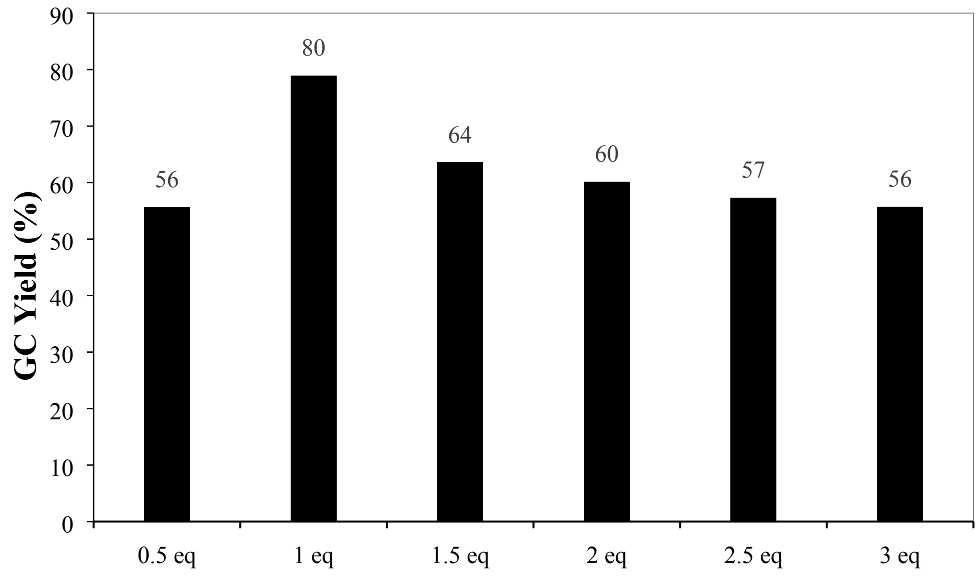

Fig. S4. Effect of base loadings (K_2CO_3) on reaction yield

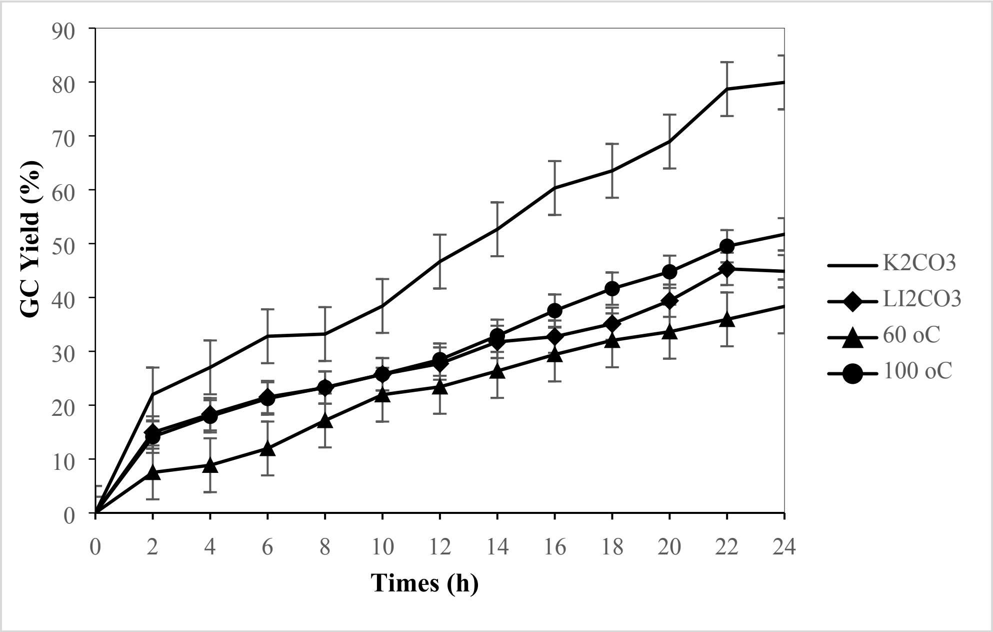

Fig. S5. Kinetic studies of reaction parameters

Table S2. Optimization for phenoxylation

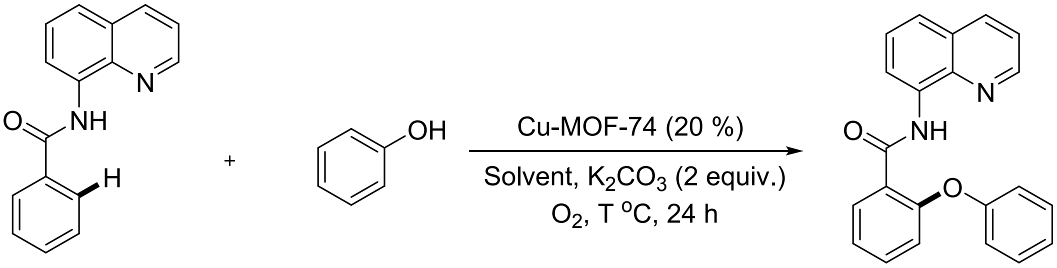

Entry	Base	Ratio C-H/phenol	Temperature (°C)	Solvent	Yield (%)[b]
1	K_2CO_3	1	80	DMF	45
2	Li_2CO_3	1	80	DMF	42 (leached)
3	Cs_2CO_3	1	80	DMF	22
4	K_2CO_3	0.5	80	DMF	68
5	K_2CO_3	1.5	80	DMF	36
6	K_2CO_3	0.5	60	DMF	22
**7**	**K_2CO_3**	**0.5**	**100**	**DMF**	**88**
8	K_2CO_3	0.5	120	DMF	91
9	K_2CO_3	0.5	100	Pyridine	<5
10	K_2CO_3	0.5	100	NMP	77

11	K_2CO_3	0.5	100	Dioxane	35
12	K_2CO_3	0.5	100	Toluene	<5
13[c]	K_2CO_3	0.5	100	DMF	80
14[d]	K_2CO_3	0.5	100	DMF	56
15[e]	K_2CO_3	0.5	100	DMF	82

[a] 0.2 mmol scale, base (02 equiv.), 24 h, 2 mL volume of solvent. [b]GC yields. [c] reaction at 12 hours. [d]reaction with 1mL of solvent. [e]reaction with 3.0 mL of solvent. freaction in 12 h..

N-(quinolin-8-yl)benzamide

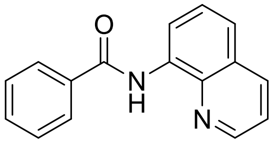

^{1}H NMR (500 MHz, $CDCl_3$, ppm): δ_H10.76 (s, 1H),8.95 (dd, *J* = 7.5, 1.0 Hz, 1H), 8.86 (dd, *J* = 4.0, 1.5 Hz, 1H), 8.20 (dd, *J* = 8.5, 1.5 Hz, 1H), 8.10- 8.09 (m, 2H), 7.62- 7.54 (m, 5H), 7.49 (dd, *J* = 8.5, 4.0 Hz, 1H).

^{13}C NMR (125 MHz, CDCl3, ppm): δ_C165.7 (C), 148.4 (CH), 138.9(C), 136.6 (CH), 135.3(C), 134.8 (C), 132.0(CH), 128.9(2 x CH), 128.2 (C), 127.7(CH), 127.5 (2 x CH), 121.8(2 x CH), 116.8(CH).

2-(Methoxy)-*N*-(quinolin-8-yl)benzamide

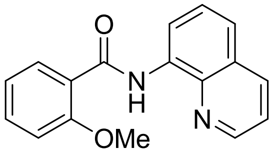

^{1}H NMR (500 MHz, $CDCl_3$, ppm): δ_H12.34 (s, 1H),9.04 (d, *J* = 7.5 Hz, 1H), 8.88 (m, 1H), 8.36 (dd, *J* = 7.5, 1.5 Hz, 1H), 8.17 (m, 1H), 7.59 (t, *J* = 8.5 Hz, 1H), 7.51 (m, 2H), 7.46 (m, 1H), 7.15 (t, *J* = 7.5 Hz, 1H), 7.09 (dd, *J* = 8.0, 1.0 Hz, 1H),4.21 (s, 3H).

^{13}C NMR (125 MHz, CDCl3, ppm): δ_C163.8 (C), 157.9 (C), 148.4 (CH), 139.4 (C), 136.4 (CH), 135.9 (C), 133.3 (CH), 132.5 (CH), 128.3 (C), 127.7 (CH), 122.5 (C), 121.6 (CH), 121.6(CH), 121.4 (CH), 117.5 (CH), 111.8 (CH), 56.27(O-CH_3).

2-(Ethoxy)-*N*-(quinolin-8-yl)benzamide

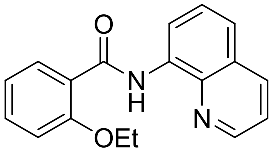

^{1}H NMR (500 MHz, $CDCl_3$, ppm): δ_H12.08 (s, 1H),9.09 (dd, *J* = 8.0, 1.5 Hz, 1H), 8.83(dd, *J* = 4.5, 1.5 Hz, 1H), 8.36 (dd, *J* = 7.5, 1.5 Hz, 1H), 8.17(dd, *J* = 8.0, 1.5 Hz, 1H), 7.59 (t, *J* = 8.0 Hz, 1H), 7.49 (m, 3H), 7.12 (t, *J* = 7.5, 1.0 Hz,1H), 7.07 (t, *J* = 8.5 Hz, 1H), 4.39 (q, *J* = 2.0 Hz, 2H), 4.21 (t, *J* = 2.0 Hz, 3H).

^{13}C NMR (125 MHz, CDCl3, ppm): δ_C164.0 (C), 157.2 (C), 147.8(CH), 139.3 (C), 136.3(CH), 135.9 (C), 133.1(CH), 132.6(CH), 128.1 (C), 127.6(CH), 122.5 (C), 121.5(CH), 121.4(CH), 121.1(CH), 117.8(CH), 112.3(CH), 65.2 (O-CH_2), 15.1(CH_3).

2-(Isopropoxy)-*N*-(quinolin-8-yl)benzamide

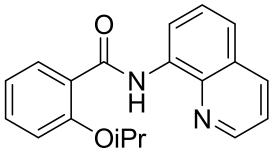

^{1}H NMR (500 MHz, $CDCl_3$, ppm): δ_H12.05 (s, 1H),9.11 (dd, *J* = 8.0, 1.0 Hz, 1H), 8.85 (dd, *J* = 4.0, 1.5 Hz, 1H), 8.35 (dd, *J* = 8.0, 1.5 Hz, 1H), 8.17(d, *J* = 8.5Hz, 1H), 7.60-7.45 (m, 4H), 7.10 (t, *J* = 7.5 Hz, 2H), 4.93 (sept, *J* = 6.0 Hz, 1H), 1.61 (d, *J* = 6.0 Hz, 6H).

2-(nButoxy)-*N*-(quinolin-8-yl)benzamide

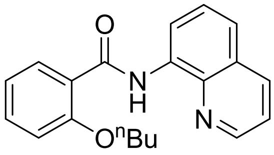

^{1}H NMR (500 MHz, $CDCl_3$, ppm): δ_H12.04 (s, 1H),9.13 (d, *J* = 8.0 Hz, 1H), 8.89-8.86(m, 1H), 8.38 (dd, *J* = 8.0, 2.0 Hz, 1H), 8.23- 8.18(m, 1H), 7.63- 7.54 (m, 4H), 7.15 (t, *J* = 7.5 Hz, 1H), 7.12- 7.09 (m, 1H), 4.35- 4.31 (m, 2H), 2.22- 2.16 (m, 2H), 1.57- 1.52 (m, 2H), 0.95 (t, *J* = 7.5 Hz, 3H).

2-(5'-Hexenoxy)-*N*-(quinolin-8-yl)benzamide

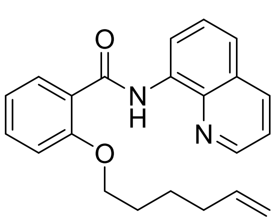

^{1}H NMR (500 MHz, $CDCl_3$, ppm): δ_H11.98 (s, 1H),9.10 (dd, *J* = 7.5, 1.0 Hz, 1H), 8.84- 8.83 (m, 1H), 8.34 (dd, *J* = 8.0, 2.0 Hz, 1H), 8.21- 8.18(m, 1H), 7.49- 7.47 (m, 4H), 7.12 (t, *J* = 7.5 Hz, 1H), 7.07 (dd, *J* = 8.0, 2.0 Hz, 1H), 5.69 (ddt, *J* = 17.0, 10.0, 6.5 Hz, 1H), 4.94- 4.87 (m, 2H), 4.31 (t, *J* = 7.0 Hz, 2H), 2.18 (pent, *J* = 7.5 Hz,2H), 2.06 (q, *J* = 7.5 Hz, 2H), 1.59 (pent, *J* = 7.5 Hz, 2H).

2-(Hydroxyethoxy)-*N*-(quinolin-8-yl)benzamide

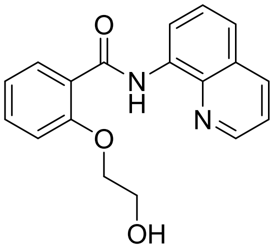

^{1}H NMR (500 MHz, $CDCl_3$, ppm): δ_H11.53 (s, 1H),9.21 (d, *J* = 7.5 Hz, 1H), 8.93- 8.92 (m, 1H), 8.40 (d, *J* = 8.0 Hz, 1H), 8.25-8.23(m, 1H), 7.64- 7.60 (m, 1H), 7.58- 7.55 (m, 1H), 7.53- 7.50(m, 2H), 7.18- 7.14 (m, 1H), 7.08- 7.06 (m, 1H), 5.17 (br, 1H), 4.35- 4.34 (m, 2H), 4.24- 4.23 (m, 2H).

^{13}C NMR (125 MHz, CDCl3, ppm): δ_C 164.0 (C), 157.1 (C),148.7 (CH), 138.9 (C), 137.8 (CH),135.3 (C), 133.6 (CH), 133.1 (CH), 128.7 (C), 127.9 (CH), 122.3 (CH), 122.0 (C), 121.7 (CH), 121.6 (CH), 119.2 (CH), 112.1 (CH), 71.4 (CH_2), 60.5 (CH_2).

2-(Phenoxy)-*N*-(quinolin-8-yl)benzamide

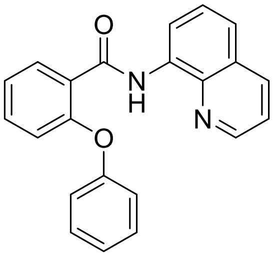

^{1}H NMR (500 MHz, $CDCl_3$, ppm): δ_H12.23 (s, 1H),8.99 (dd, *J* = 8.0, 1.5 Hz, 1H), 8.57 (dd, *J* = 4.0, 1.5 Hz, 1H), 8.39 (dd, *J* = 8.0, 1.5 Hz, 1H), 8.11(dd, *J* = 8.5, 1.5 Hz, 1H),

7.57 (t, J = 8.0 Hz, 1H), 7.50(dd, J = 8.0, 1.0 Hz, 1H), 7.46 (ddd, J = 8.5, 7.5, 2.0 Hz, 1H), 7.41 (m, 2H), 7.37 (dd, J = 8.5, 4.0 Hz, 1H), 7.27 (m, 3H), 7.19 (tt, J = 7.5, 1.0 Hz, 1H),7.03(dd, J = 8.5, 1.0 Hz, 1H).

^{13}C NMR (125 MHz, CDCl3, ppm): δ_C163.3 (C), 156.1 (C), 155.6 (C), 148.2 (CH), 139.3 (C), 136.2 (CH), 135.6 (C), 133.1 (CH), 132.5 (CH), 130.0 (2 x CH), 128.1 (C), 127.6 (CH), 125.1 (C), 124.4(CH), 123.8(CH), 121.7(CH), 121.6(CH), 119.8(2 x CH), 118.6 (CH), 117.3 (CH).

2-(4-Methylphenoxy)-*N*-(quinolin-8-yl)benzamide

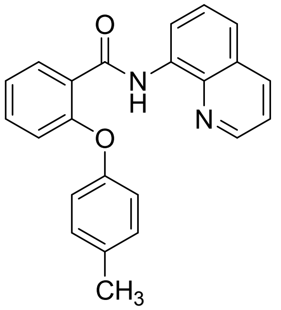

^{1}H NMR (500 MHz, $CDCl_3$, ppm): δ_H12.37 (s, 1H),9.09 (d, J = 7.5 Hz, 1H), 8.67 (d, J = 3.0 Hz, 1H), 8.48 (dd, J = 8.0, 1.5 Hz, 1H), 8.20(d, J = 8.0 Hz, 1H), 7.66 (t, J=8.0 Hz, 1H), 7.58 (d, J = 8.0 Hz, 1H), 7.54- 7.51 (m, 1H), 7.46 (dd, J = 8.0, 4.0 Hz, 1H), 7.34 (d,J = 5.0 Hz,1H), 7.32- 7.30 (m, 2H), 7.25 (d, J = 8.5 Hz, 2H), 7.09 (d, J = 8.0 Hz, 1H), 2.46 (s, 3H).

^{13}C NMR (125 MHz, CDCl3, ppm): δ_C 163.4 (C), 156.1 (C), 153.6 (C), 148.2 (CH), 139.3 (C), 136.2 (CH), 135.7 (C), 134.1 (C), 133.0 (CH), 132.5 (CH),130.5 (2 x CH), 128.1 (C), 127.6 (CH), 124.7 (C),123.4 (CH), 121.7 (CH), 121.6 (CH), 119.9 (2 x CH), 118.0 (CH), 117.3 (CH), 20.9 (CH_3).

2-(2-Methylphenoxy)-*N*-(quinolin-8-yl)benzamide

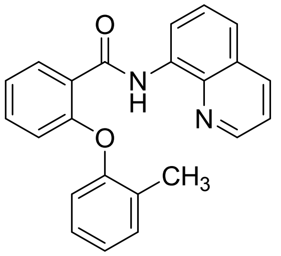

^{1}H NMR (500 MHz, $CDCl_3$, ppm): δ_H12.27 (s, 1H),9.03 (dd, J = 8.0, 1.5 Hz, 1H), 8.46 (dd, J = 4.0, 1.5 Hz, 1H), 8.39 (dd, J = 7.5, 1.5 Hz, 1H), 8.11(dd, J = 8.0, 1.5 Hz, 1H), 7.58 (t, J=8 Hz, 1H), 7.50 (m, 1H), 7.39 (ddd, J = 8.0, 7.5, 1.5 Hz, 1H), 7.34 (m, 2H), 7.24 (m, 2H), 7.18 (td, J = 7.5, 1.0 Hz, 1H), 7.10 (dd, J = 8.0, 1.0 Hz, 1H), 2.43 (s, 3H).

^{13}C NMR (125 MHz, CDCl3, ppm): δ_C 163.5 (C), 162.1 (C), 156.4 (C), 153.4 (C), 148.1 (CH), 139.3 (C), 136.2 (CH), 133.1 (CH), 132.5 (CH), 131.7 (CH), 130.8 (C),128.1 (C), 127.6 (2 x CH), 125.2 (CH), 123.9(C), 122.9 (CH), 121.7 (CH), 121.6 (CH), 120.9 (CH), 117.4 (CH), 116.4 (CH), 16.5 (CH_3).

2-(3-Methylphenoxy)-*N*-(quinolin-8-yl)benzamide

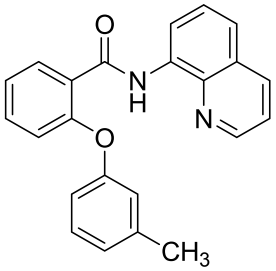

^{1}H NMR (500 MHz, $CDCl_3$, ppm): δ_H12.42 (s, 1H),9.18 (dd, J = 8.0, 1.0 Hz, 1H), 8.76(dd, J = 4.0, 1.5 Hz, 1H), 8.57 (dd, J = 7.5, 1.5 Hz, 1H), 8.29(dd, J = 8.0, 1.5 Hz, 1H), 7.80- 7.72 (m, 2H), 7.68- 7.62 (m, 2H), 7.55(dd, J = 8.0, 4.0 Hz, 1H), 7.49- 7.43(m, 2H), 7.26- 7.25(m, 1H), 7.22- 7.18 (m, 2H), 2.55 (s, 3H).

2-(2-Methoxylphenoxy)-*N*-(quinolin-8-yl)benzamide

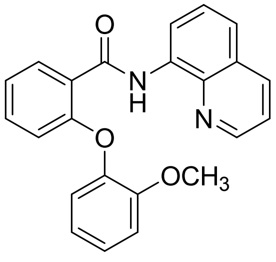

^{1}H NMR (500 MHz, $CDCl_3$, ppm): δ_H12.27 (s, 1H),9.01 (dd, J = 7.5, 1.0 Hz, 1H), 8.42 (dd, J = 4.0, 1.5 Hz, 1H), 8.33 (dd, J = 8.0, 2.0 Hz, 1H), 8.10(dd, J = 8.0, 1.5 Hz, 1H), 7.58 (t, J=8 Hz, 1H), 7.49 (dd, J = 8.0, 1.0 Hz, 1H), 7.37 (ddd, J = 8.0, 7.5, 1.5 Hz, 1H), 7.32 (m, 2H), 7.26 (m, 1H), 7.19 (td, J = 7.5, 1.0 Hz, 1H), 7.06 (m, 2H), 6.79 (dd, J = 8.5, 1.0 Hz, 1H), 3.75 (s, 3H).

^{13}C NMR (125 MHz, CDCl3, ppm): δ_C 163.7 (C), 156.8 (C), 152.1 (C), 148.1 (CH), 143.8 (C),139.4 (C), 136.1 (CH), 135.8 (C), 132.8 (CH), 132.2 (CH), 128.1 (C),127.6 (CH), 126.2 (CH), 123.7(C), 122.9 (CH), 122.7 (CH), 121.5 (2 x CH), 121.4 (CH), 117.3 (CH), 115.9 (CH), 114.3 (CH), 56.2 (O-CH_3).

2-(4-Methoxylphenoxy)-*N*-(quinolin-8-yl)benzamide

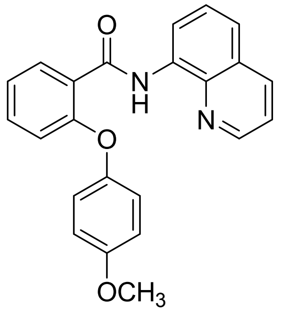

^1H NMR (500 MHz, $CDCl_3$, ppm): δ_H12.31 (s, 1H),9.01 (d, *J* = 7.5 Hz, 1H), 8.56 (d, *J* = 3.0 Hz, 1H), 8.38 (d, *J* = 8.0 Hz, 1H), 8.12 (d, *J* = 8.0 Hz, 1H), 7.58 (t, *J* = 8.0 Hz, 1H), 7.50 (d,*J* = 8.0 Hz,1H), 7.44- 7.41 (m, 1H), 7.37 (dd, *J* = 8.0, 4.0 Hz, 1H), 7.25- 7.21 (m, 3H), 6.96 (dd, *J* = 9.0, 3.0 Hz, 3H), 3.84 (s, 3H).

^{13}C NMR (125 MHz, CDCl3, ppm): δ_C 163.3 (C), 156.5 (CH), 148.9 (C), 148.1 (CH), 139.2 (C), 136.1 (CH), 135.6 (C), 132.9 (CH), 132.3 (CH), 128.0 (C), 127.5 (CH), 124.2 (C), 123.0 (CH), 121.5 (CH), 121.4 (CH), 121.3 (2 x CH), 117.2 (CH), 117.1 (CH), 115.0 (2 x CH), 55.7 (C).

2-(4-Iodophenoxy)-*N*-(quinolin-8-yl)benzamide

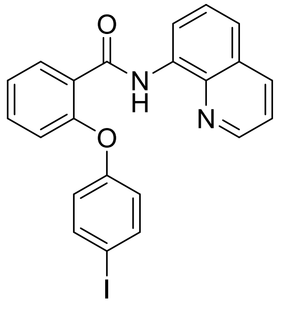

^1H NMR (500 MHz, $CDCl_3$, ppm): δ_H12.04 (s, 1H),8.97 (dd, *J* = 7.5, 1.0 Hz, 1H), 8.59(dd, *J* = 4.0, 1.5 Hz, 1H), 8.38 (dd, *J* = 8.0, 1.5 Hz, 1H), 8.13 (dd, *J* = 8.0, 1.0 Hz, 1H), 7.69 (m, 2H), 7.57(t, *J* = 8.0Hz, 1H), 7.49(m, 2H), 7.40(dd, *J* = 8.0, 4.0 Hz, 1H), 7.31(m, 1H), 7.02 (m, 3H).

^{13}C NMR (125 MHz, CDCl3, ppm): δ_C163.0 (C), 156.3 (C), 154.7 (C), 148.2 (CH), 139.2 (C), 139.0(2 x CH), 136.3 (CH), 135.4(C), 133.2(CH), 132.7 (2 x CH), 128.1 (C), 127.6 (CH), 125.5 (C), 121.8(CH), 121.7(3 x CH), 118.9(CH), 117.3 (CH), 87.4 (C).

2-(4-Nitrophenoxy)-*N*-(quinolin-8-yl)benzamide

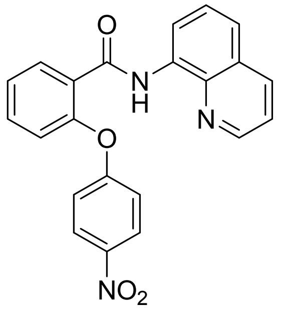

^{1}H NMR (500 MHz, $CDCl_3$, ppm): δ_H11.67 (s, 1H),8.93 (dd, J = 7.5, 1.0 Hz, 1H), 8.66(d, J = 4.0Hz, 1H), 8.38 (dd, J = 7.5, 1.5 Hz, 1H), 8.23(d, J = 8.0 Hz, 2H), 8.15(d, J = 8.5 Hz, 1H), 7.56 (m, 3H), 7.44 (m, 2H),7.26 (m, 2H), 7.15(d, J = 8.0 Hz, 1H).

^{13}C NMR (125 MHz, CDCl3, ppm): δ_C162.5 (C), 162.1 (C), 152.8 (C), 148.2 (CH), 143.6 (C), 139.0 (C), 136.5 (CH), 135.0 (C), 133.5(CH), 132.9 (CH), 128.2 (C), 127.6 (CH), 127.0 (C), 126.1 (CH), 126.0(2 x CH), 122.1(CH), 121.8 (CH), 120.9 (CH), 118.3(2 x CH),117.4 (CH).

4-Chloro-*N*-(quinolin-8-yl)benzamide

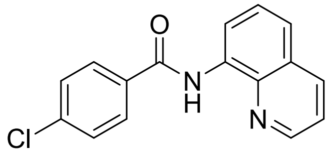

^{1}H NMR (500 MHz, $CDCl_3$, ppm): δ_H10.72 (s, 1H),8.90 (d, J = 7.0 Hz, 1H), 8.85 (s, J =1H), 8.20 (d, J = 7.5 Hz, 1H), 8.03(d, J = 8.0 Hz, 2H), 7.62- 7.51 (m, 5H).

^{13}C NMR (125 MHz, CDCl3, ppm): δ_C164.5 (C), 148.4 (CH), 138.8 (C), 138.3 (C), 136.7 (CH), 134.4 (C), 133.6 (C), 129.2 (2 x CH), 128.9 (2 x CH), 128.2 (C), 127.6 (CH), 122.1 (CH), 121.9 (CH), 116.9 (CH).

4-Chloro-2-ethoxy-*N*-(quinolin-8-yl)benzamide

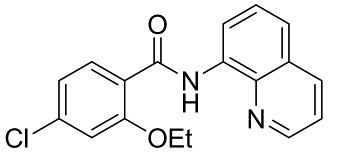

^{1}H NMR (500 MHz, $CDCl_3$, ppm): δ_H11.96 (s, 1H),9.06 (d, *J* = 7.5 Hz, 1H), 8.83- 8.81 (m, 1H), 8.29 (d, *J* = 8.5 Hz, 1H), 8.17(d, *J* = 8.5 Hz, 1H), 7.59 (t, *J* = 8.0 Hz, 1H), 7.53 (d, *J* = 8.0 Hz, 1H), 7.46 (dd, *J* = 8.0, 4.0 Hz, 1H), 7.10 (dd, *J* = 7.5, 2.0 Hz, 1H), 7.06- 7.05 (m, 1H), 4.36 (q, *J* = 7.0 Hz, 2H), 1.77 (t, *J* = 7.0 Hz, 3H).

^{13}C NMR (125 MHz, CDCl3, ppm): δ_C163.1 (C), 157.7 (C), 147.9 (CH), 139.4 (C), 138.9 (C), 136.4 (CH), 135.8 (C), 133.9 (CH), 128.2 (C), 127.7 (CH), 121.9 (CH), 121.6 (CH), 121.5 (CH), 121.2 (C), 117.9 (CH), 113.0 (CH).

4-Methoxy-*N*-(quinolin-8-yl)benzamide

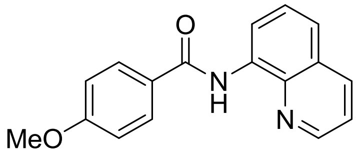

^{1}H NMR (500 MHz, $CDCl_3$, ppm): δ_H10.73 (s, 1H),8.93- 8.92 (m, 2H), 8.46- 8.41 (m, 1H), 8.19- 8.18 (m, 2H), 8.74- 8.67 (m, 3H), 7.04 (d, *J* = 8.0 Hz, 2H), 3.90 (s, 3H).

^{13}C NMR (125 MHz, CDCl3, ppm): δ_C 162.9 (C), 130.1 (CH), 128.9 (C), 122.5 (C), 121.4 (C), 114.0 (2 x CH), 55.6 (CH_3.

2-Ethoxy-4-methoxy-*N*-(quinolin-8-yl)benzamide

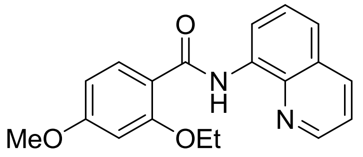

^{1}H NMR (500 MHz, $CDCl_3$, ppm): δ_H11.97 (s, 1H),9.09 (d, *J* = 7.5 Hz, 1H), 8.82 (dd, *J* = 4.0, 1.0 Hz, 1H), 8.34 (d, *J* = 9.0 Hz, 1H), 8.17(dd, *J* = 8.5, 1.5 Hz, 1H), 7.58 (t, *J* = 8.0 Hz, 1H), 7.51 (d, *J* = 8.0 Hz, 1H), 7.45 (dd, *J* = 8.5, 4.5 Hz, 1H), 6.66 (dd, *J* = 8.5, 2.0 Hz, 1H), 6.58 (d, *J* = 2.5 Hz, 1H), 4.35 (q, *J* = 7.0 Hz, 2H), 3.88 (s, 3H), 1.78 (t, *J* = 7.0 Hz, 3H).

^{13}C NMR (125 MHz, CDCl3, ppm): δ_C164.0 (C), 163.8 (C), 158.7 (C), 147.8 (CH), 139.4 (C), 136.4 (CH), 136.3 (CH), 134.4 (CH), 128.3 (C), 127.8 (C), 127.7 (CH), 121.5 (CH),

121.4 (CH), 117.8 (CH), 115.7 (C), 105.5 (CH), 99.5 (CH), 65.4 (CH_2), 55.7 (CH_3), 15.2 (CH_3).

2-Methyl-*N*-(quinolin-8-yl)benzamide

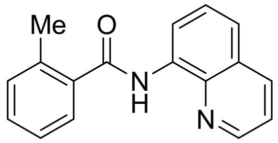

^{1}H NMR (500 MHz, $CDCl_3$, ppm): δ_H10.21 (s, 1H),8.94 (d, *J* = 7.5 Hz, 1H), 8.78 (dd, *J* = 4.0, 1.5 Hz, 1H), 8.19 (dd, *J* = 8.5, 1.0 Hz, 1H), 7.69 (d, *J* = 7.5 Hz, 1H), 7.61 (t, *J* = 8.0 Hz, 1H), 7.55 (d, *J* = 8.0 Hz, 1H), 7.46 (d, *J* = 8.0, 4.0 Hz, 1H), 7.41 (t, *J* = 7.5 Hz, 1H), 7.33 (t, *J* = 8.0 Hz, 2H), 2.61 (s, 3H).

^{13}C NMR (125 MHz, CDCl3, ppm): δ_C168.4 (C), 148.4 (CH), 138.8 (C), 136.8 (C), 136.8 (C), 136.5 (CH), 134.9(C), 131.5(CH), 130.5 (CH), 128.2(C), 127.6 (CH), 127.4(CH), 126.2(CH), 121.9 (CH), 121.8 (CH), 116.7 (CH).

2-Ethoxy-4-methyl-*N*-(quinolin-8-yl)benzamide

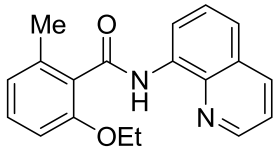

^{1}H NMR (500 MHz, $CDCl_3$, ppm): δ_H10.21 (s, 1H),9.01 (d, *J* = 7.5 Hz, 1H), 8.76 (dd, *J* = 4.0, 1.0 Hz, 1H), 8.17 (d, *J* = 8.0 Hz, 1H), 7.60 (t, *J* = 8.0 Hz, 1H), 7.54 (d,*J* = 8.5 Hz 1H), 7.44 (dd, *J* = 8.5, 4.0 Hz, 1H), 7.27 (t, *J* = 8.0 Hz, 1H), 6.88 (d, *J* = 8.0 Hz, 1H), 6.84 (d, *J* = 8.0 Hz, 1H), 4.11 (q, *J* = 7.0 Hz, 2H), 2.47 (s, 3H), 1.31 (t, *J* = 7.0 Hz, 3H).

3-Bromo-*N*-(quinolin-8-yl)benzamide

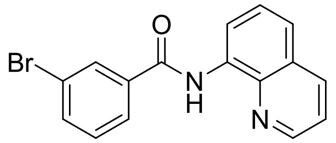

3-Bromo-2-ethoxy-*N*-(quinolin-8-yl)benzamide

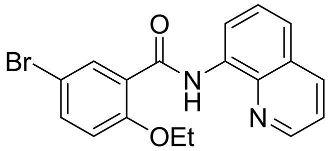

^{1}H NMR (500 MHz, $CDCl_3$, ppm): δ_H10.01 (s, 1H),9.06 (dd, *J* = 7.5, 1.0 Hz, 1H), 8.83 (dd, *J* = 4.0, 1.5 Hz, 1H), 8.47 (d, *J* = 3.0 Hz, 1H), 8.18 (dd, *J* = 8.0, 1.5 Hz, 1H), 7.61-7.54 (m, 3H), 7.47 (dd, *J* = 8.0, 4.0 Hz, 1H), 6.96 (d, *J* = 8.5 Hz, 1H), 4.37 (q, *J* = 7.0 Hz, 2H), 1.75 (t, *J* = 7.0 Hz, 3H).

^{13}C NMR (125 MHz, CDCl3, ppm): δ_C162.6 (C), 156.3 (C), 150.0 (CH), 139.4 (C), 136.4 (CH), 135.7 (CH), 135.4 (CH), 128.3 (C), 127.7 (CH), 124.4 (C), 122.0 (CH), 121.6 (CH), 118.0(CH), 114.4 (CH), 113.8 (C).

N-(quinolin-8-yl)benzamide

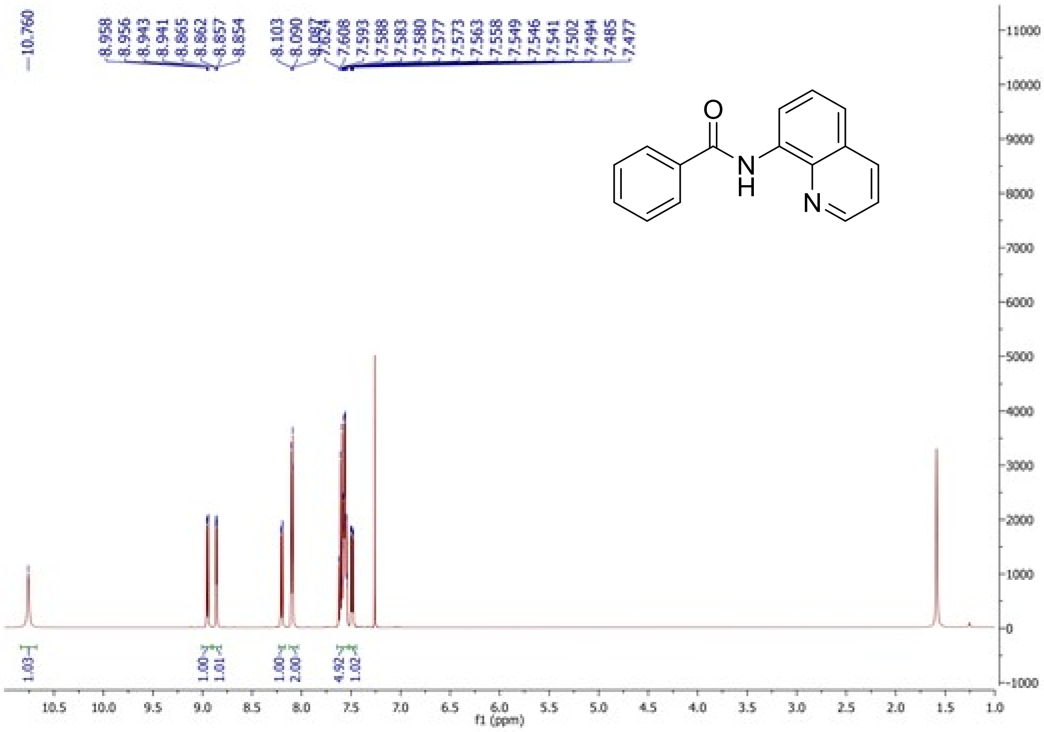

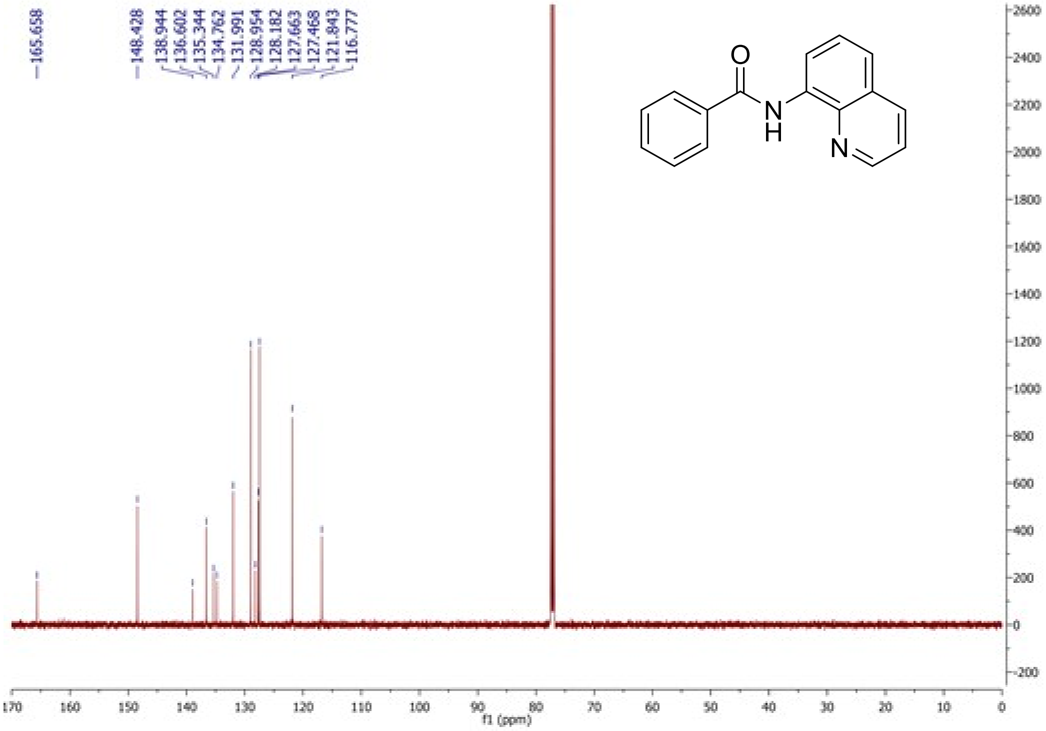

2-(Methoxy)-*N*-(quinolin-8-yl)benzamide

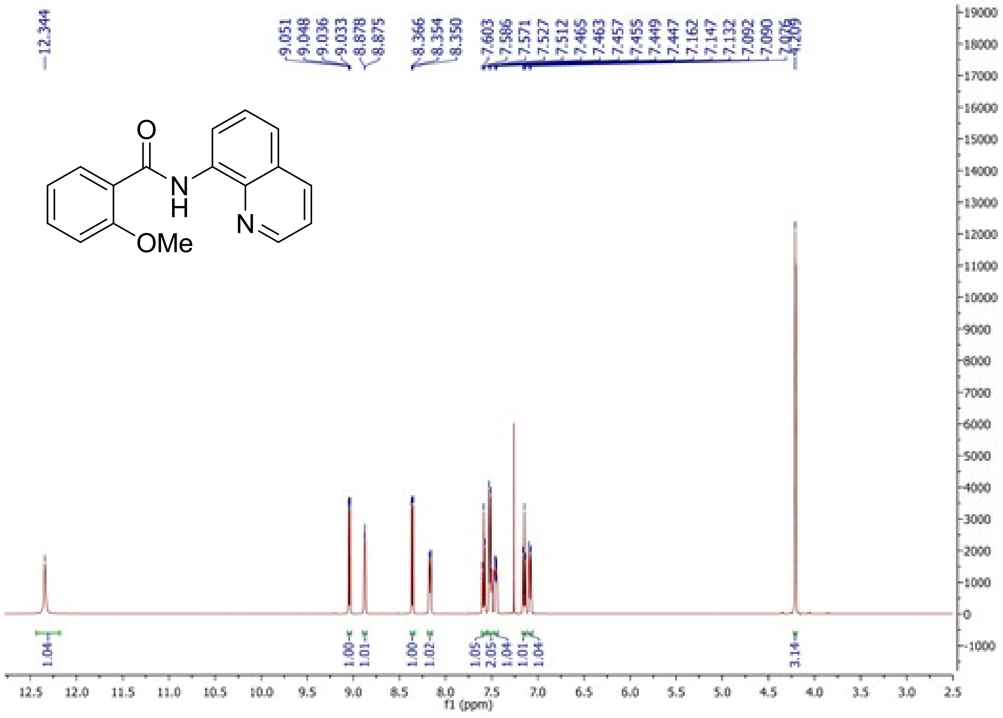

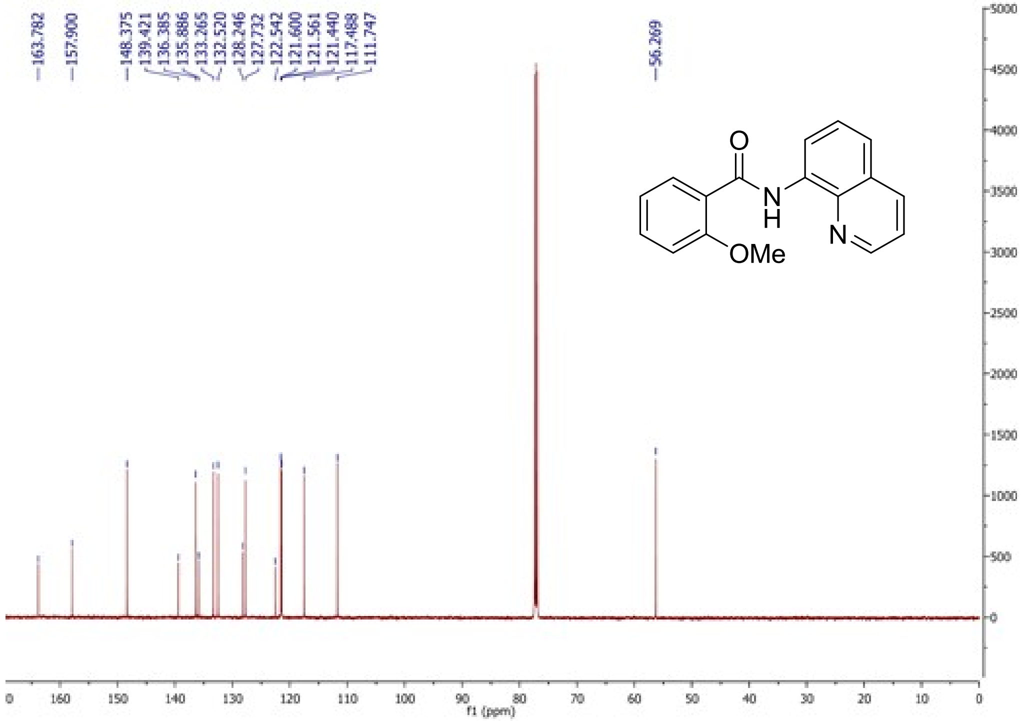

2-(Ethoxy)-*N*-(quinolin-8-yl)benzamide

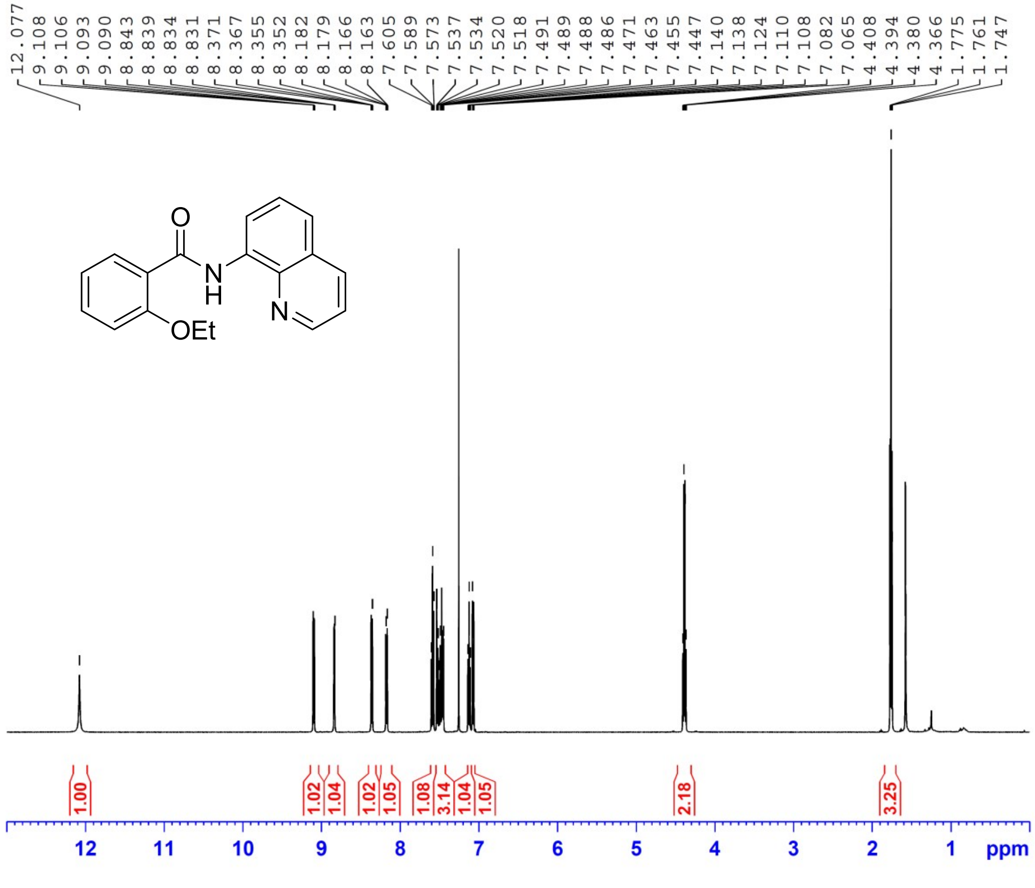

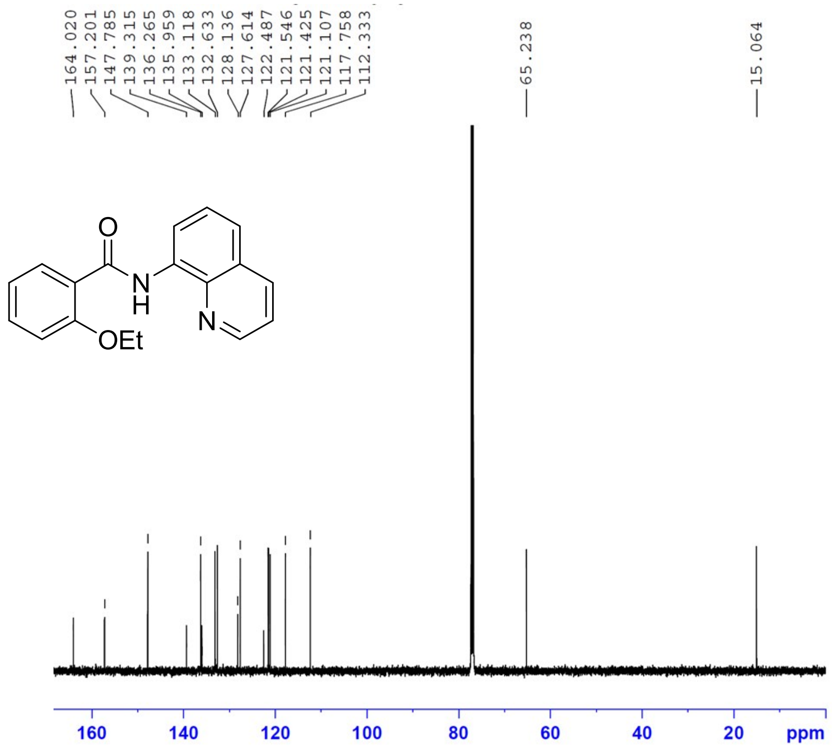

2-(Isopropoxy)-*N*-(quinolin-8-yl)benzamide

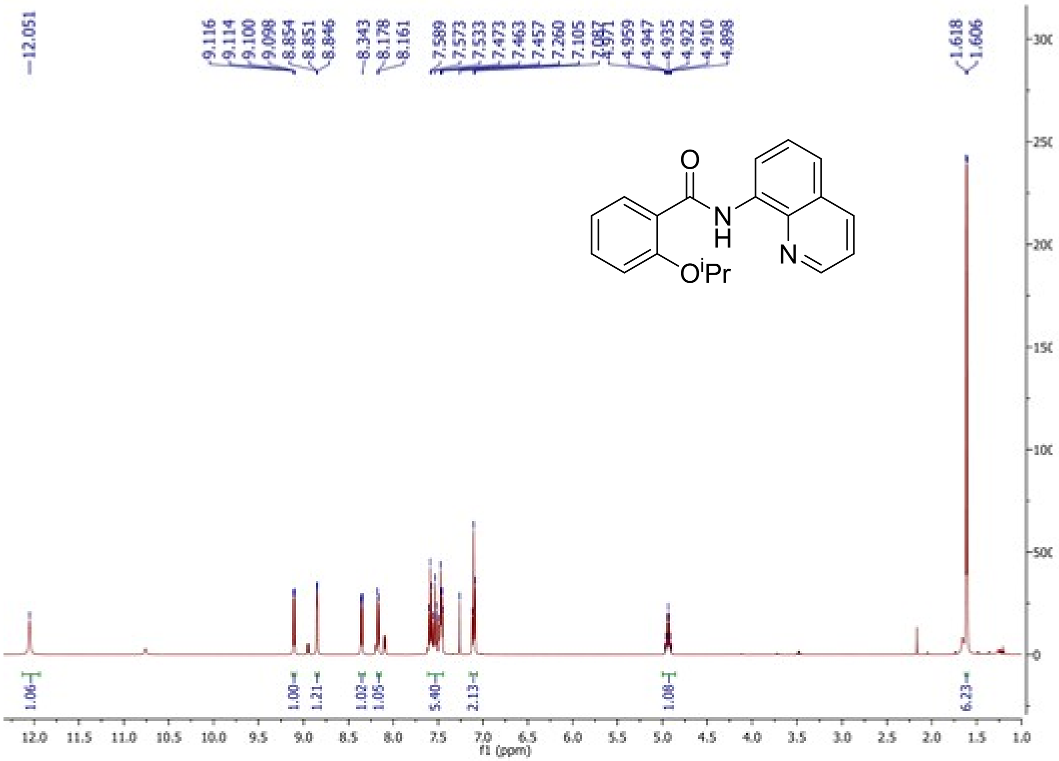

2-(nButoxy)-*N*-(quinolin-8-yl)benzamide

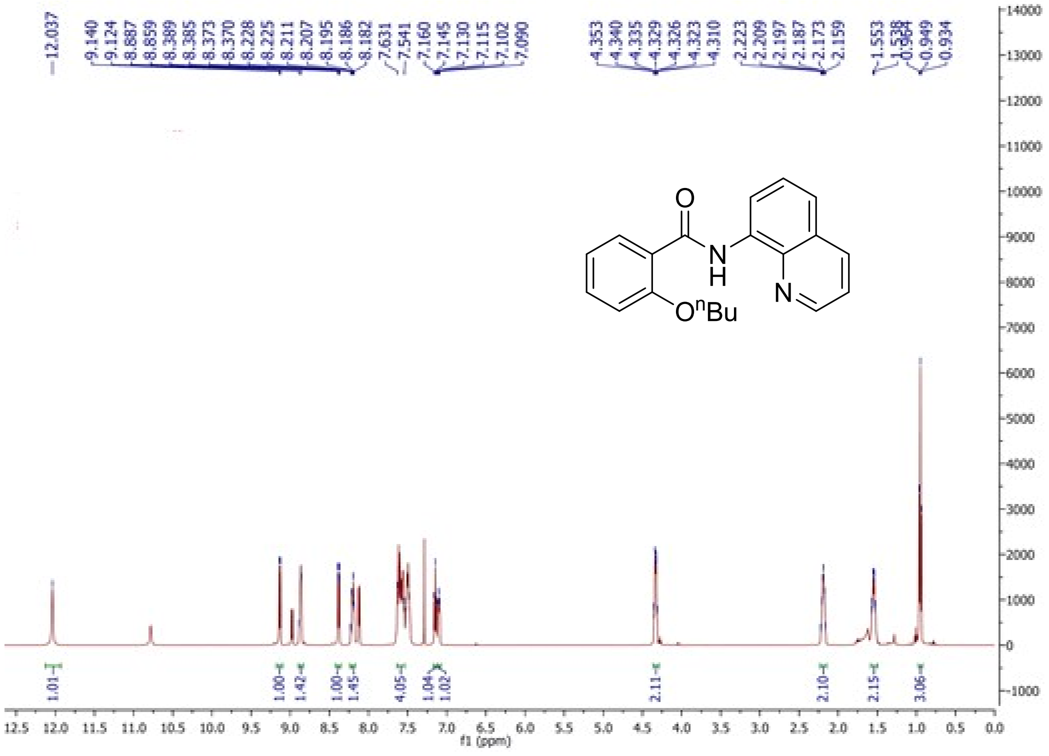

2-(5’-Hexenoxy)-*N*-(quinolin-8-yl)benzamide

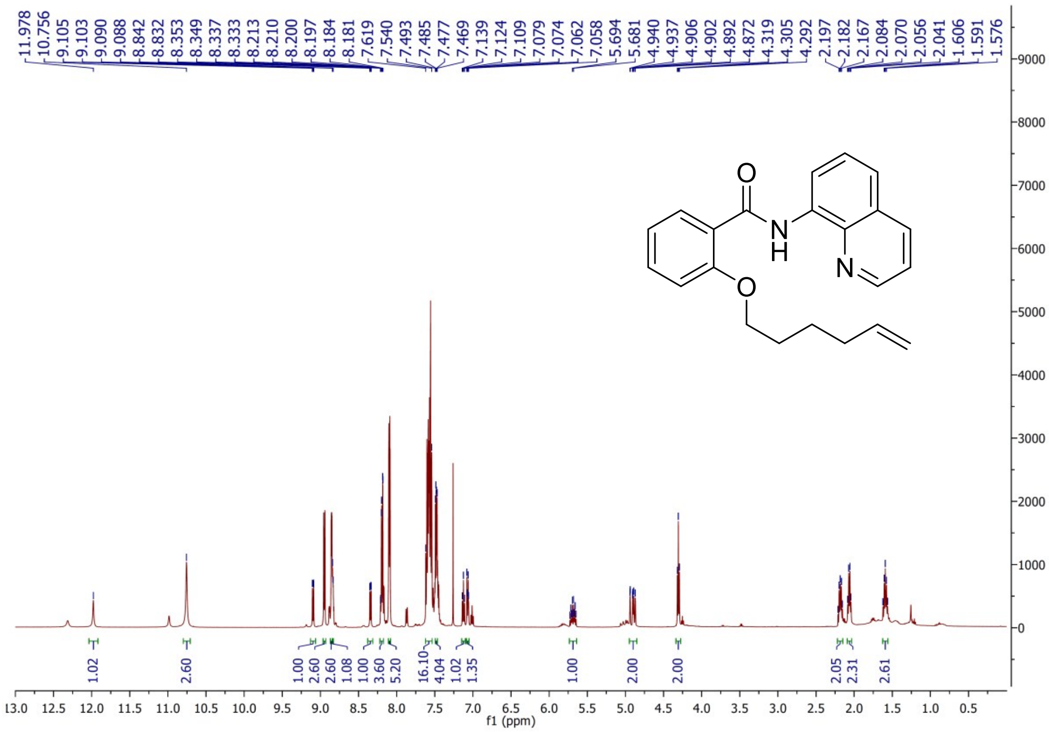

2-(Hydroxyethoxy)-*N*-(quinolin-8-yl)benzamide

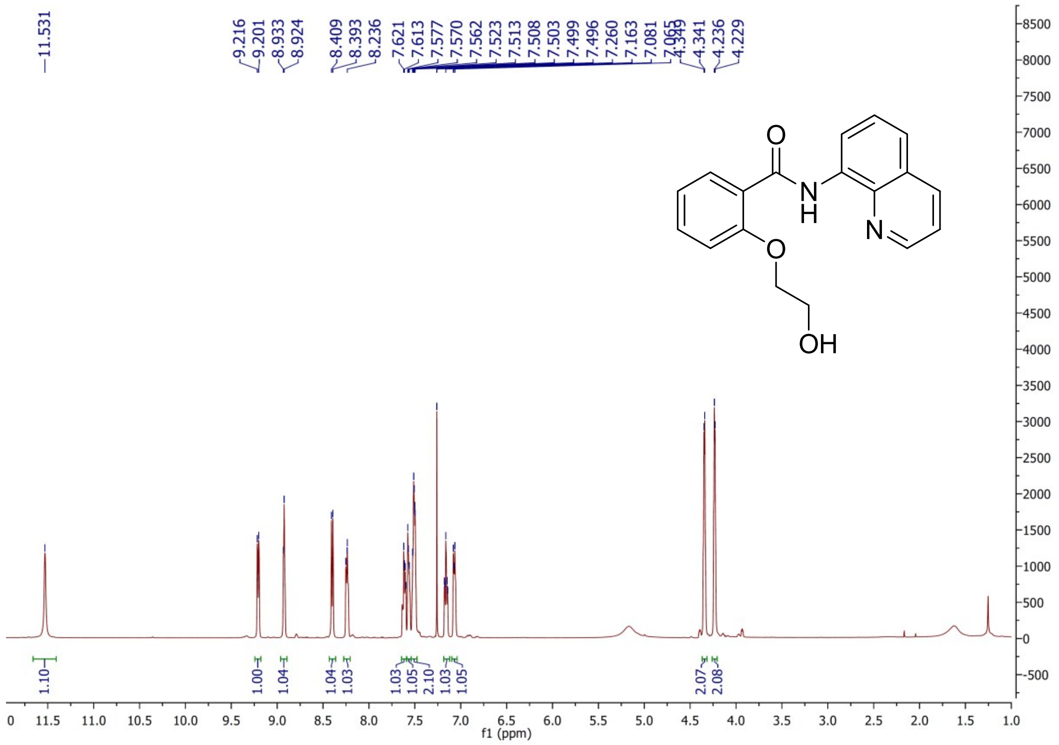

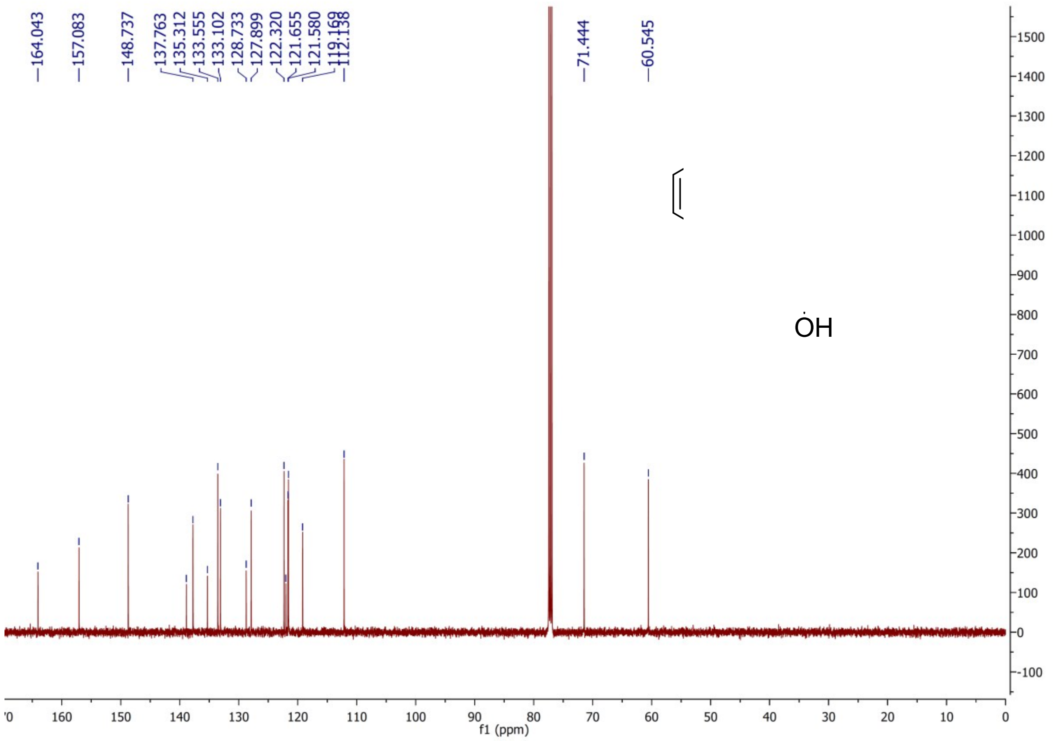

2-(Phenoxy)-*N*-(quinolin-8-yl)benzamide

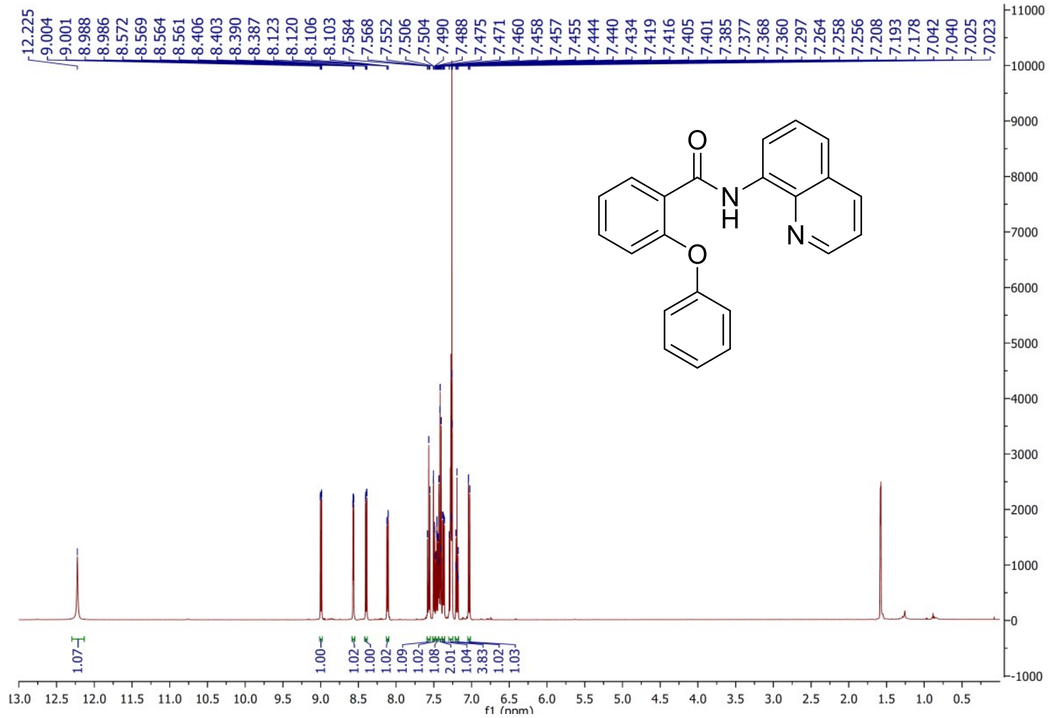

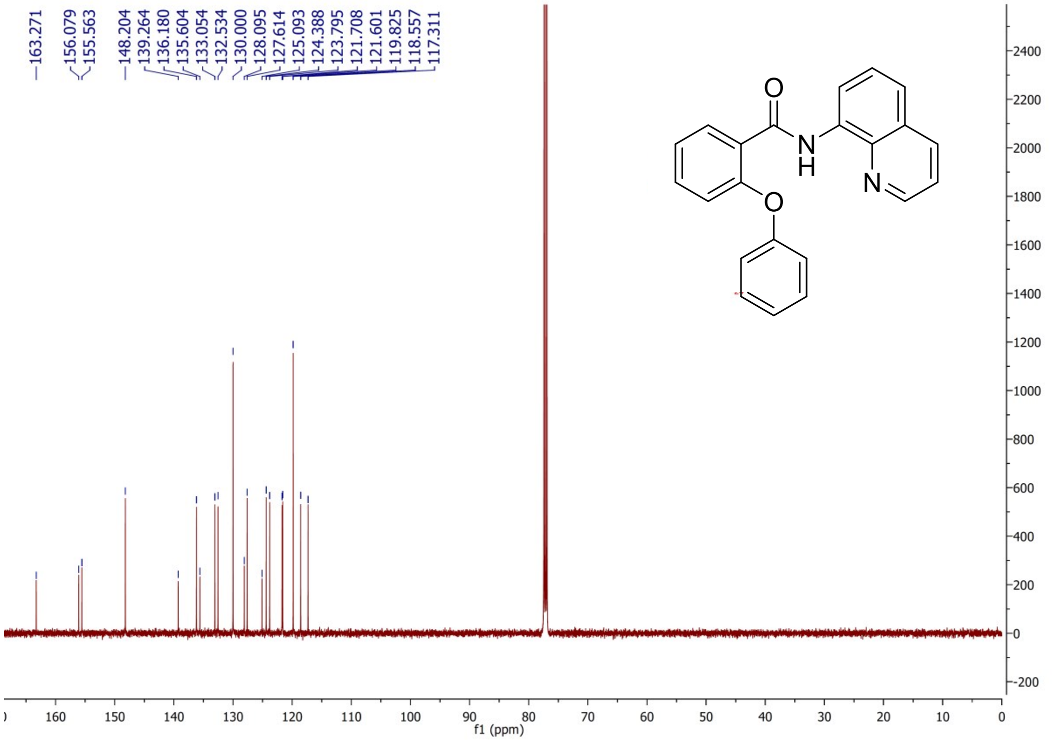

2-(4-Methylphenoxy)-*N*-(quinolin-8-yl)benzamide

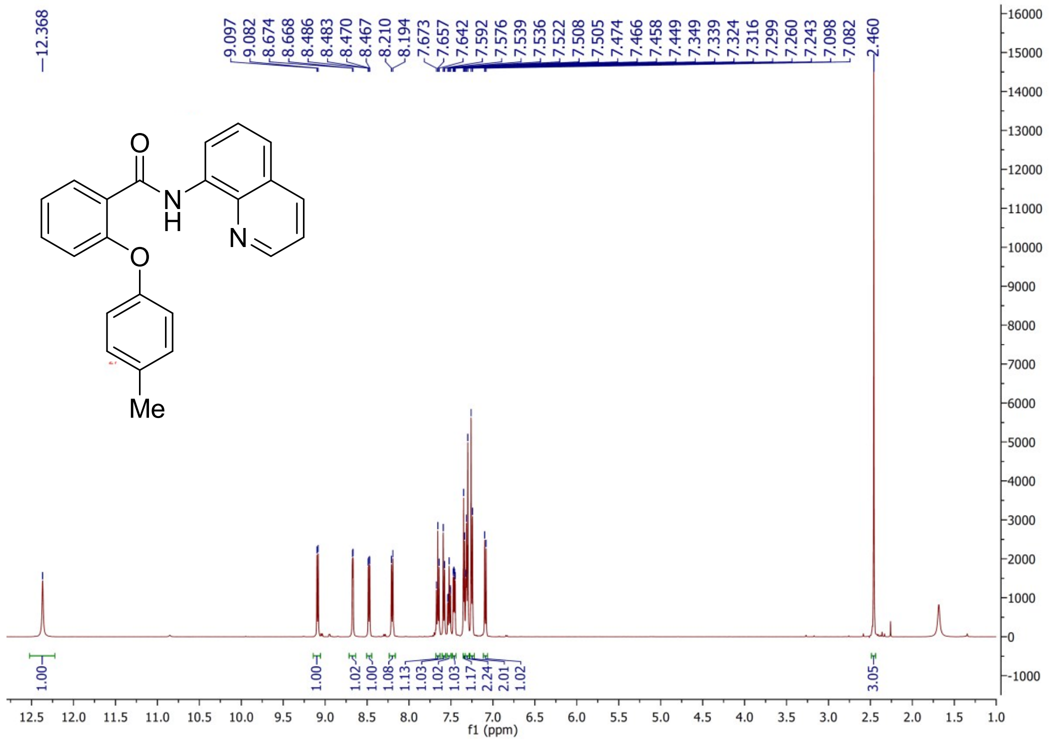

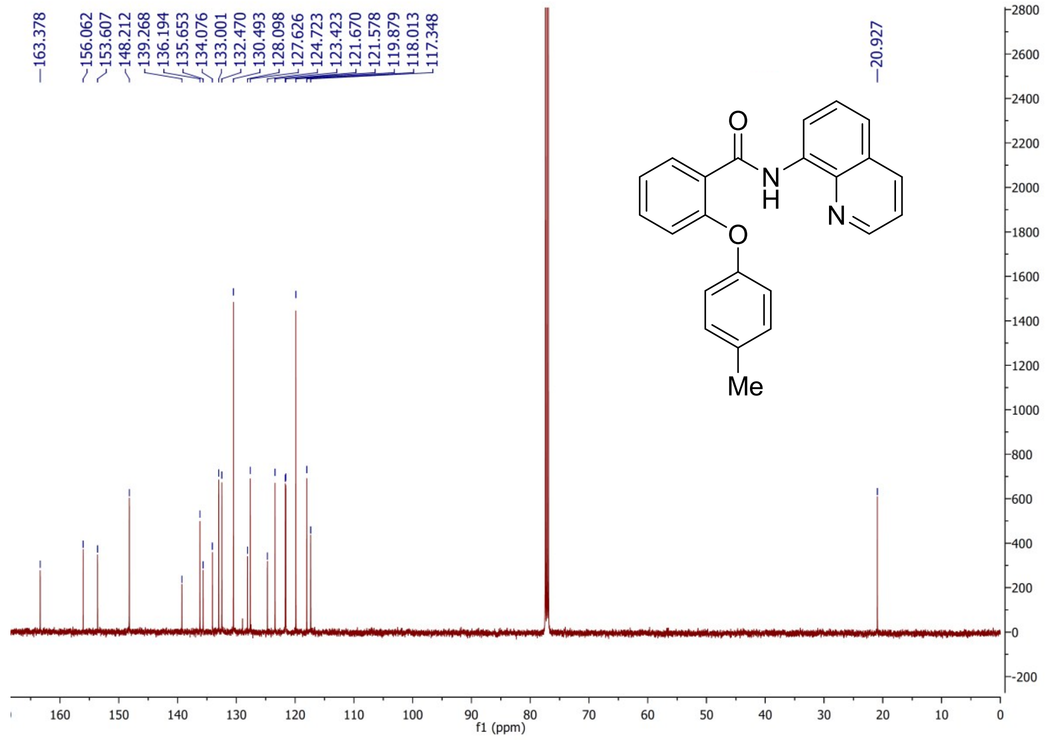

2-(2-Methylphenoxy)-*N*-(quinolin-8-yl)benzamide

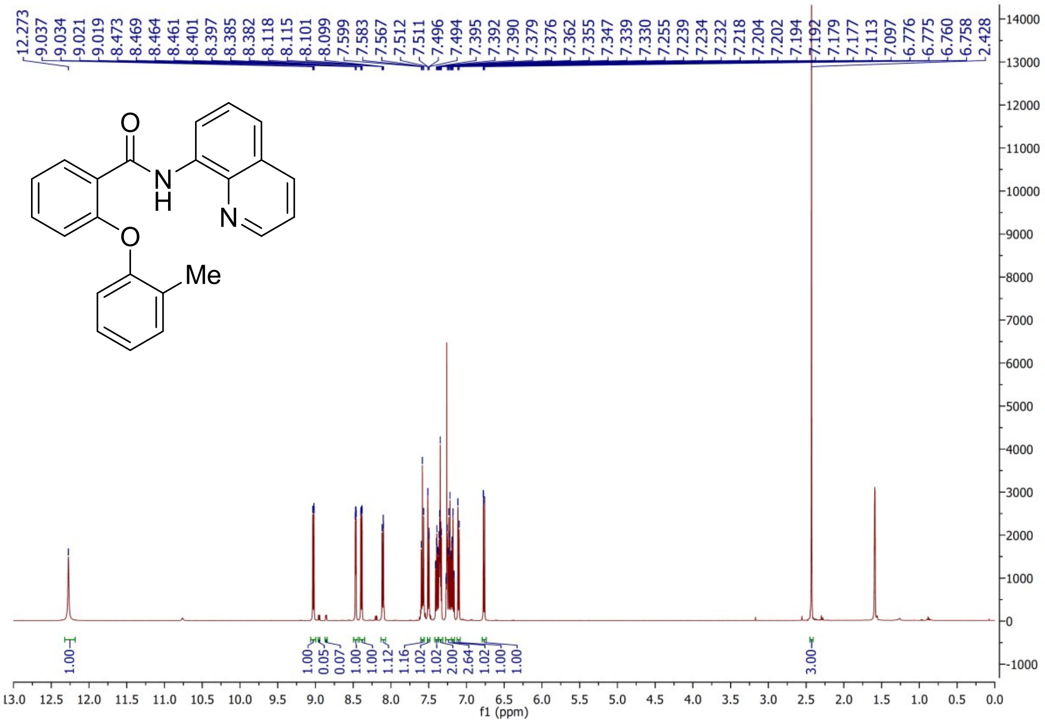

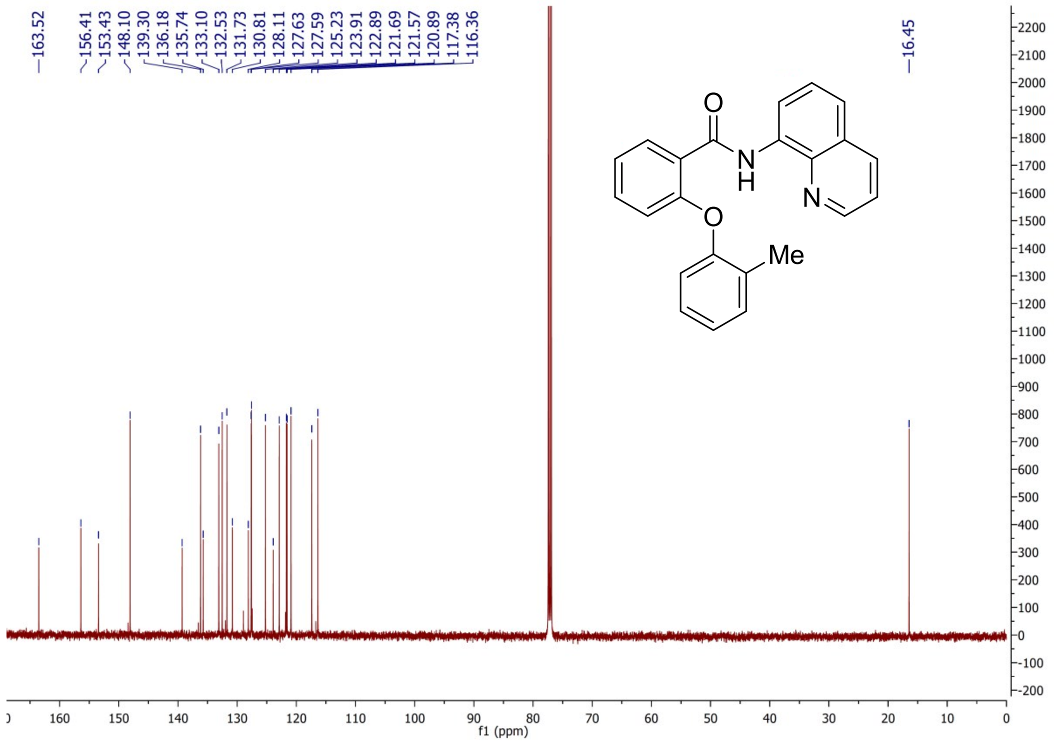

2-(3-Methylphenoxy)-*N*-(quinolin-8-yl)benzamide

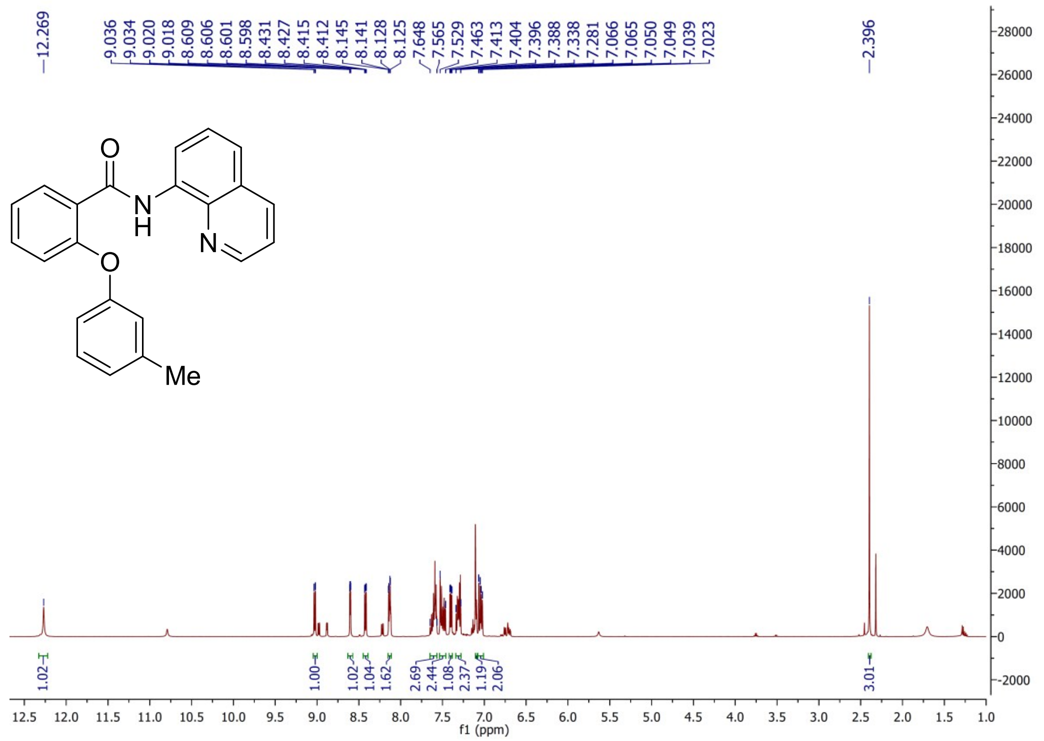

2-(2-Methoxylphenoxy)-*N*-(quinolin-8-yl)benzamide

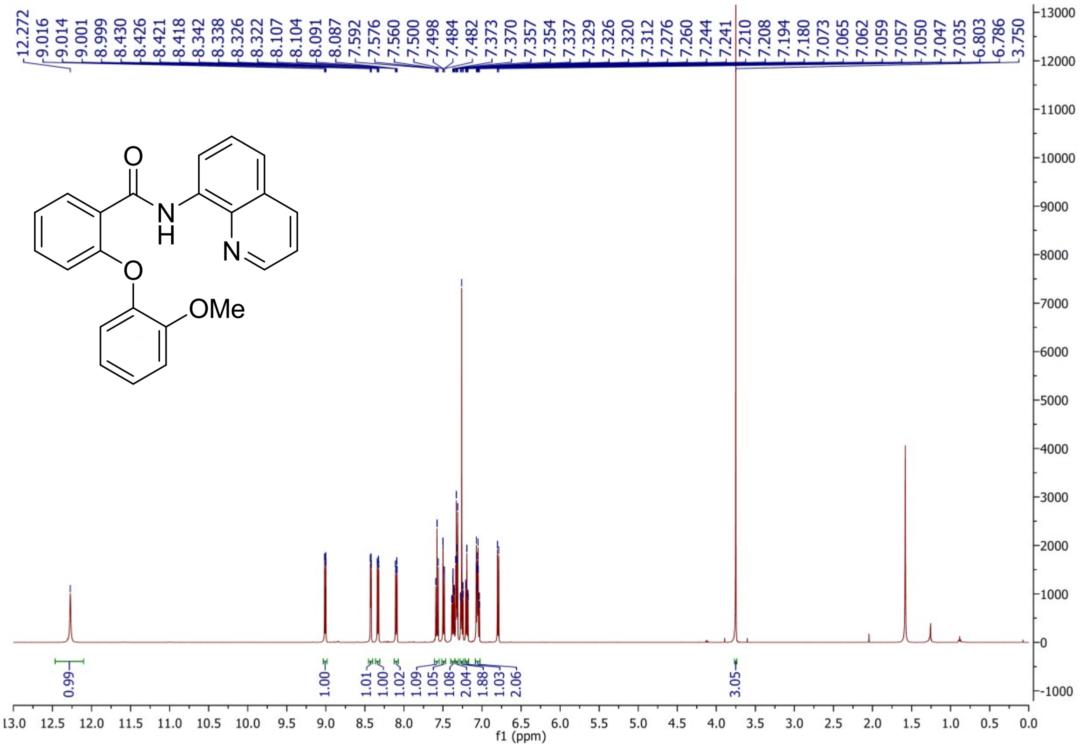

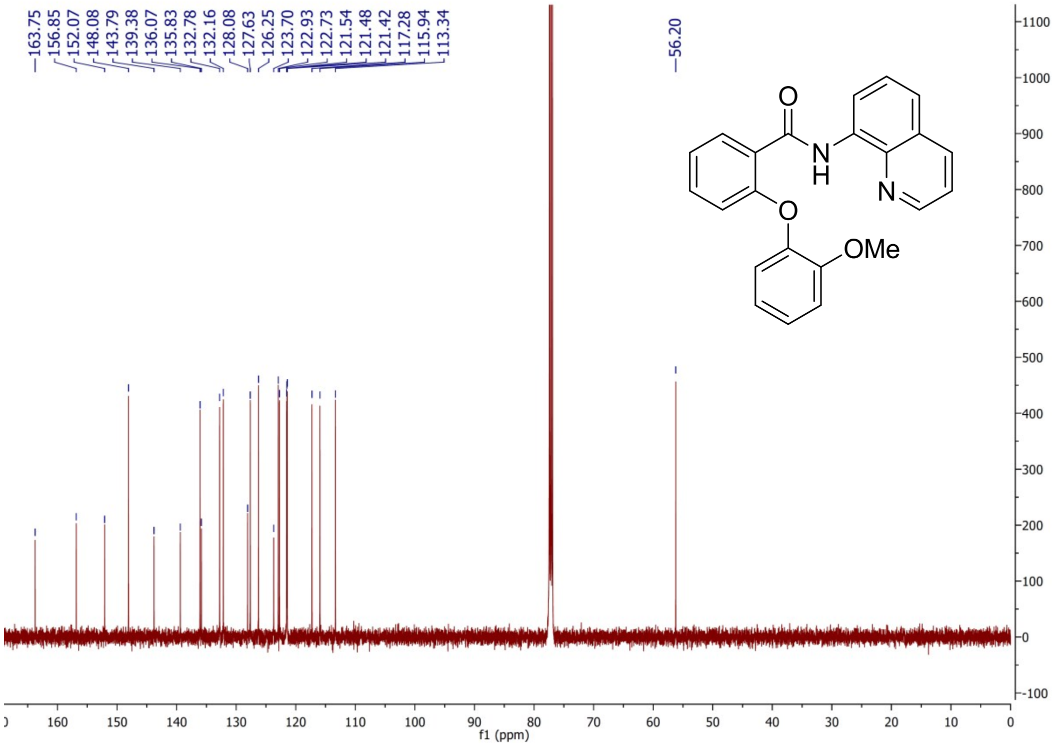

2-(4-Methoxylphenoxy)-*N*-(quinolin-8-yl)benzamide

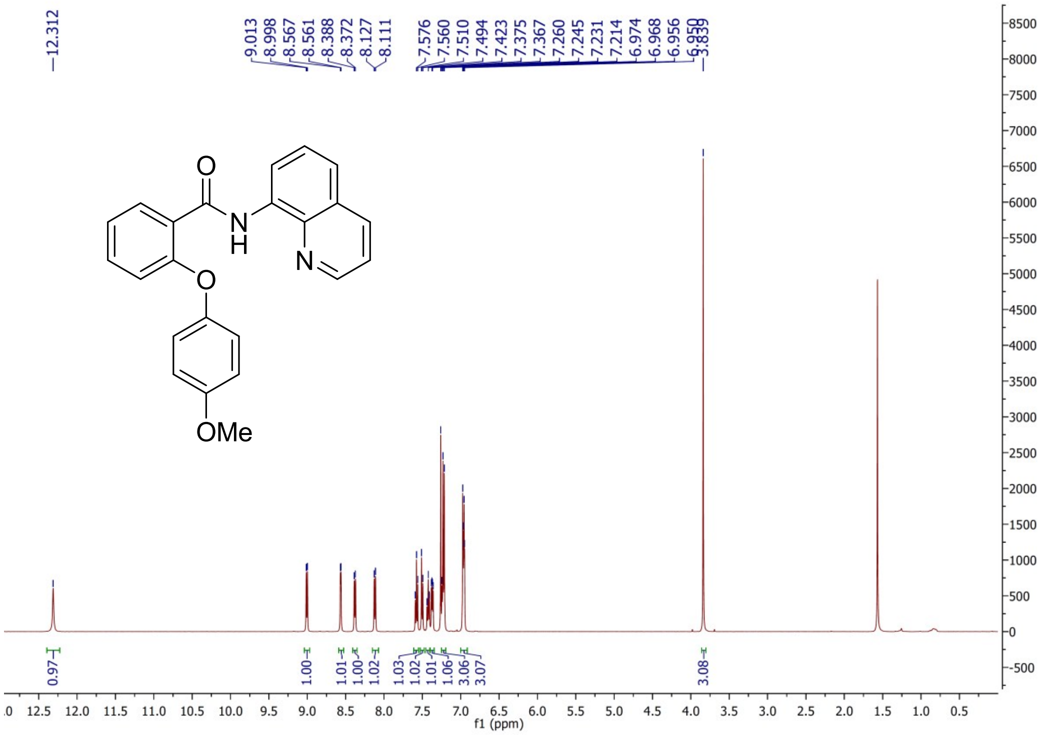

2-(4-Iodophenoxy)-*N*-(quinolin-8-yl)benzamide

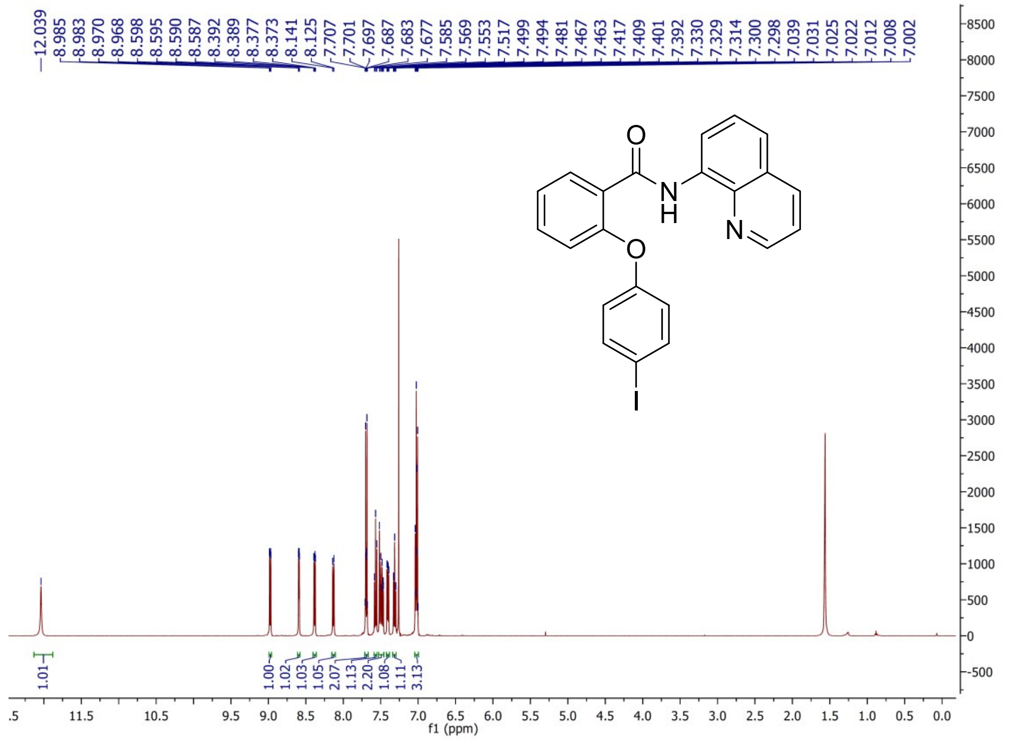

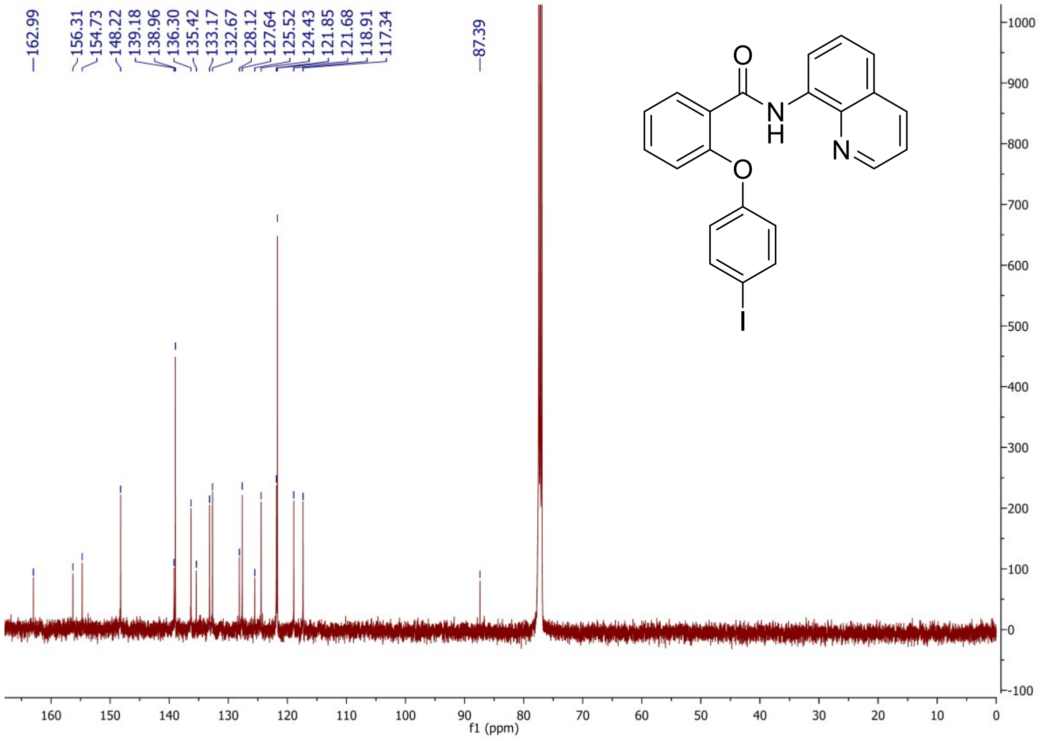

2-(4-Nitrophenoxy)-*N*-(quinolin-8-yl)benzamide

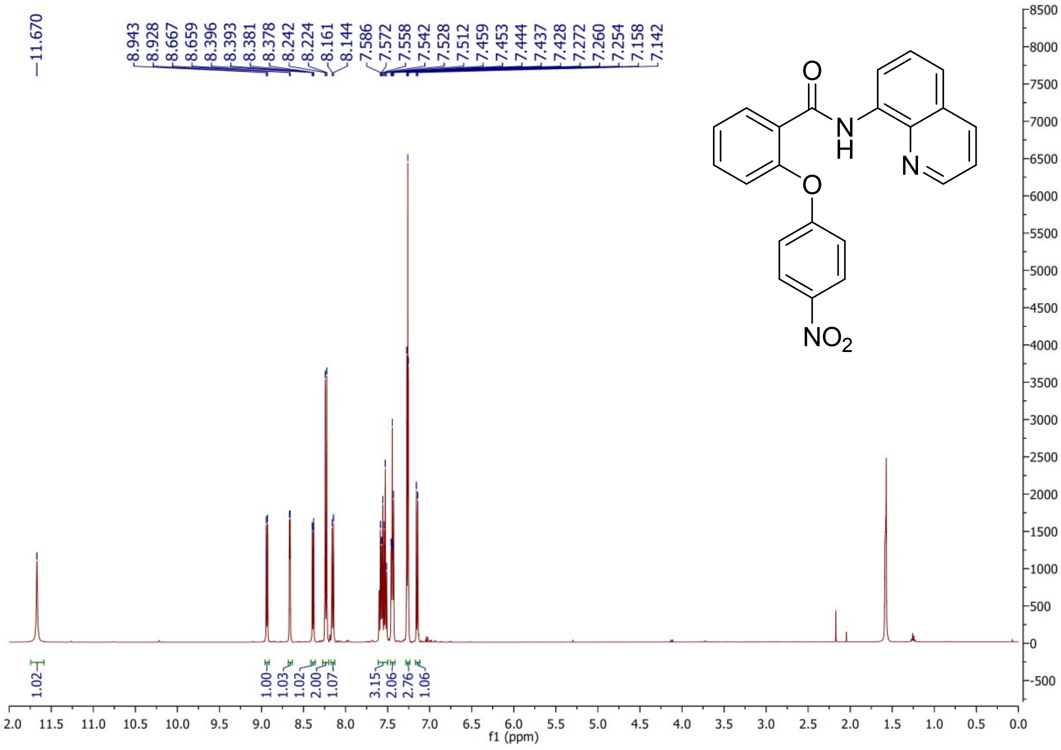

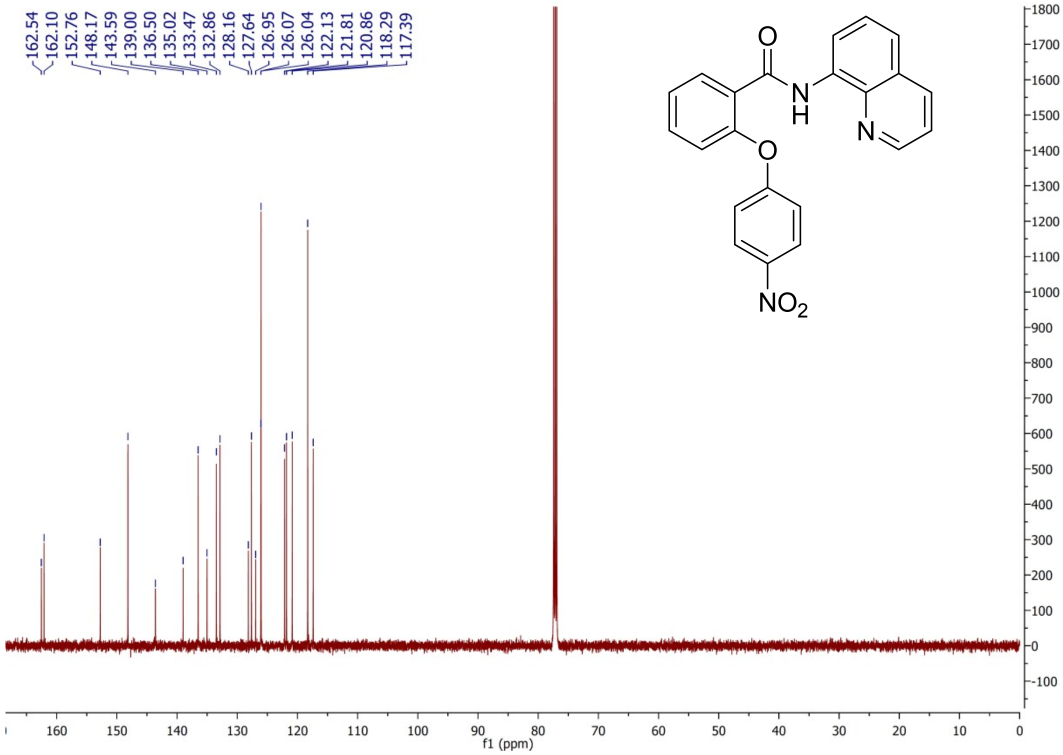

4-Chloro-*N*-(quinolin-8-yl)benzamide

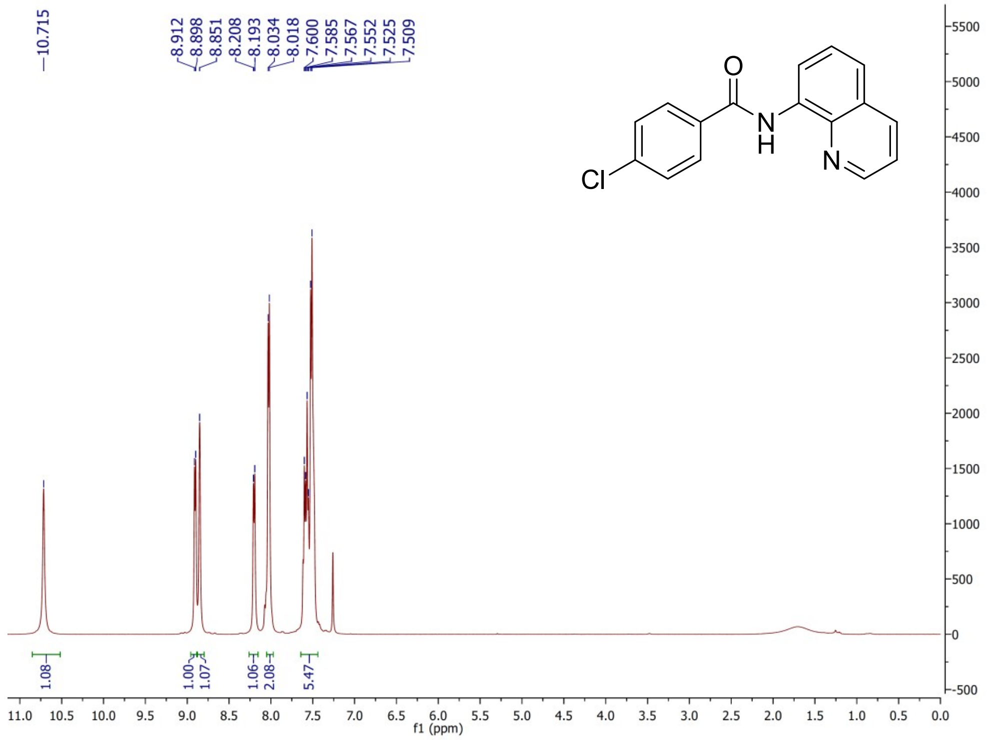

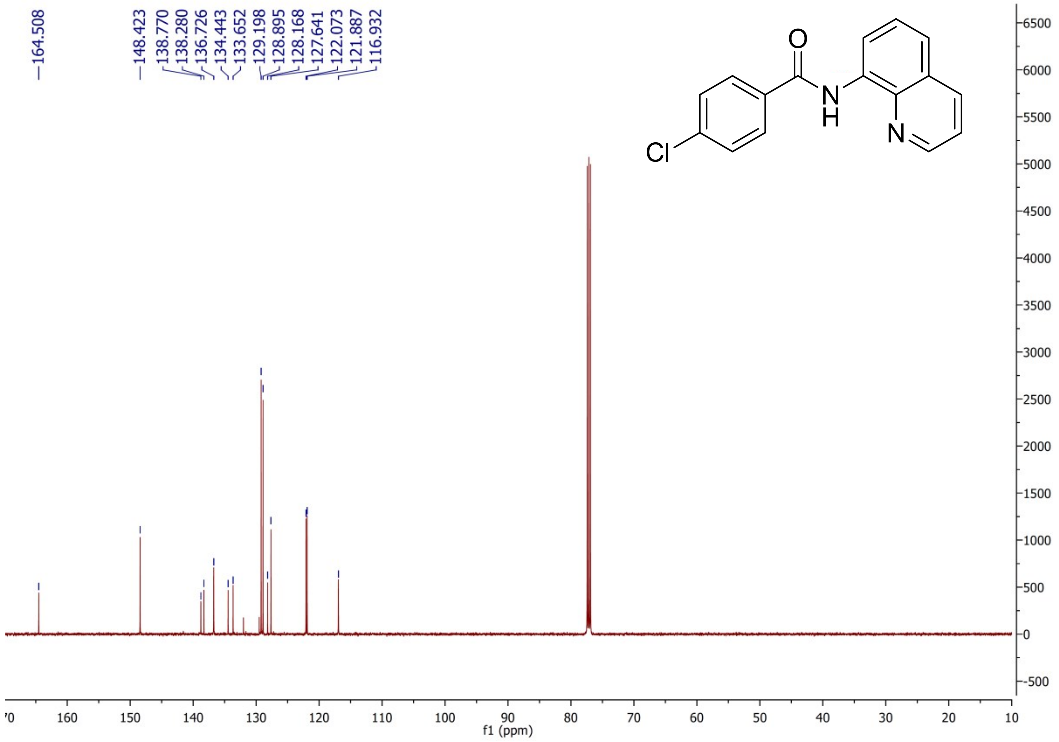

4-Chloro-2-ethoxy-*N*-(quinolin-8-yl)benzamide

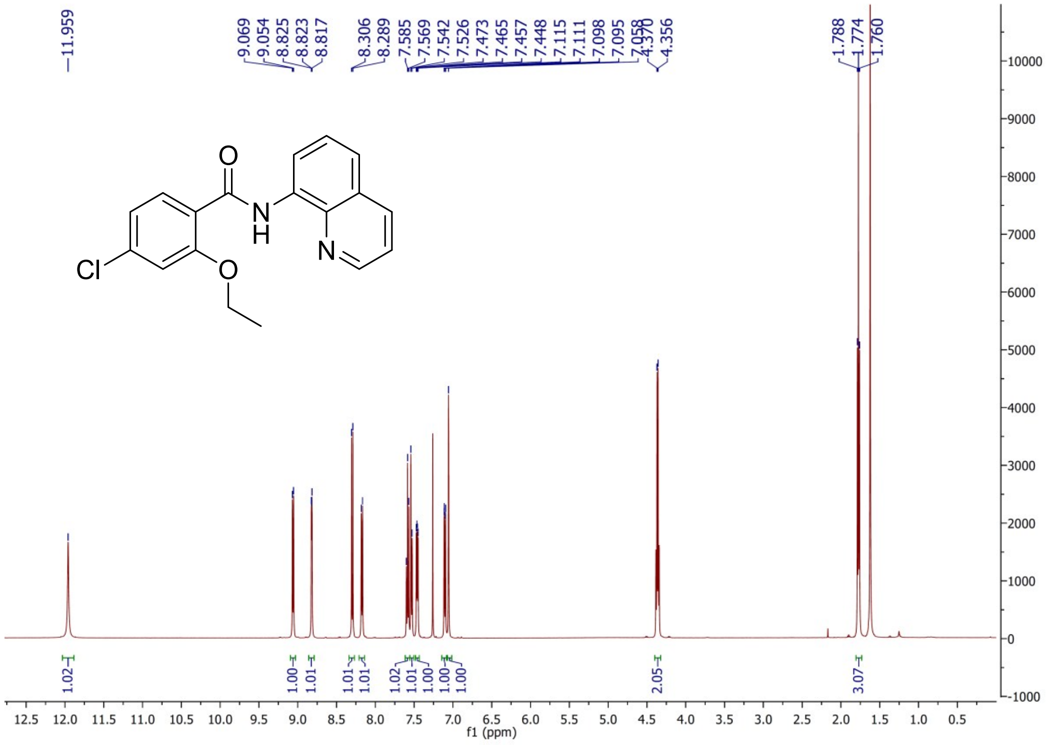

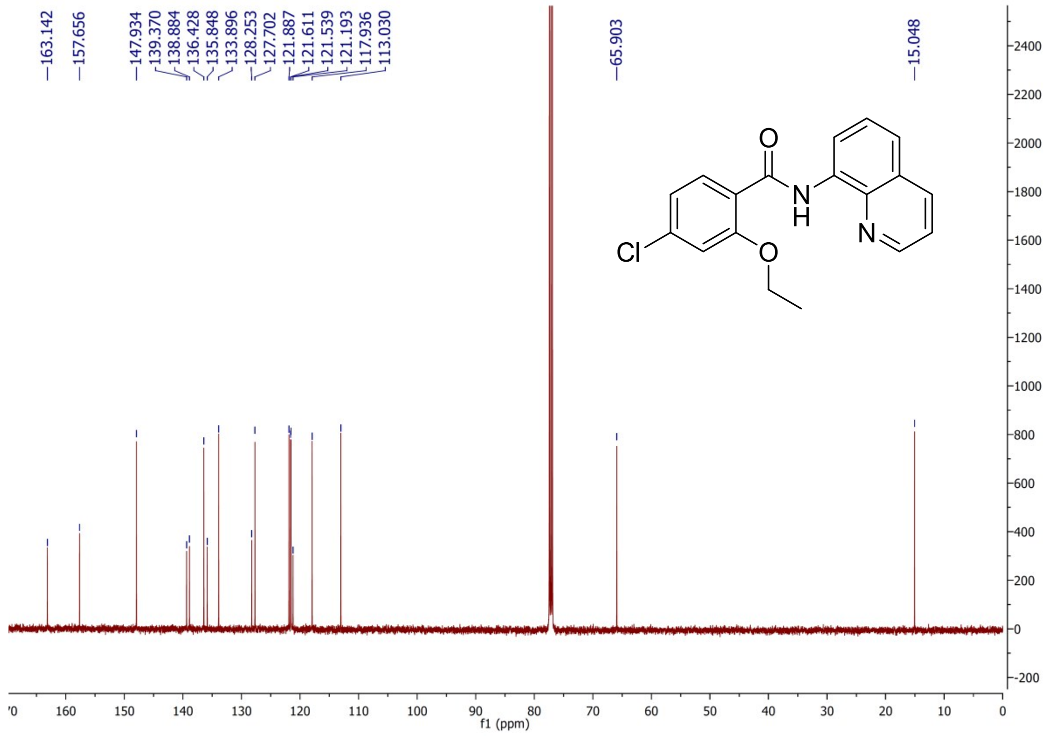

4-Methoxy-*N*-(quinolin-8-yl)benzamide

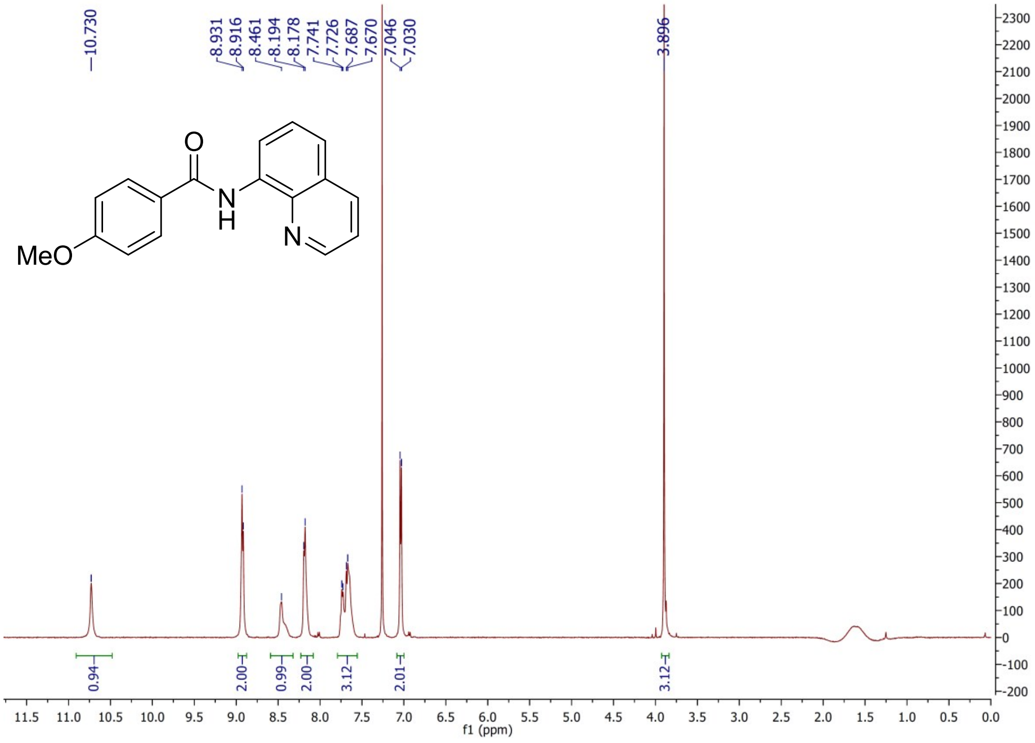

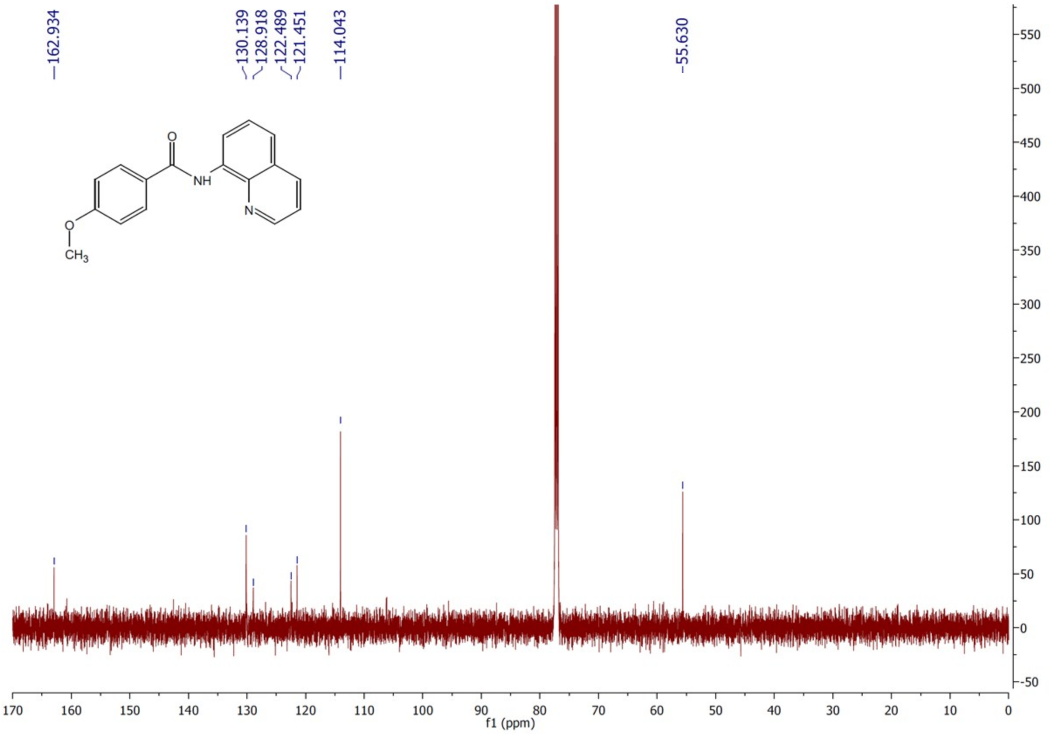

2-Ethoxy-4-methoxy-*N*-(quinolin-8-yl)benzamide

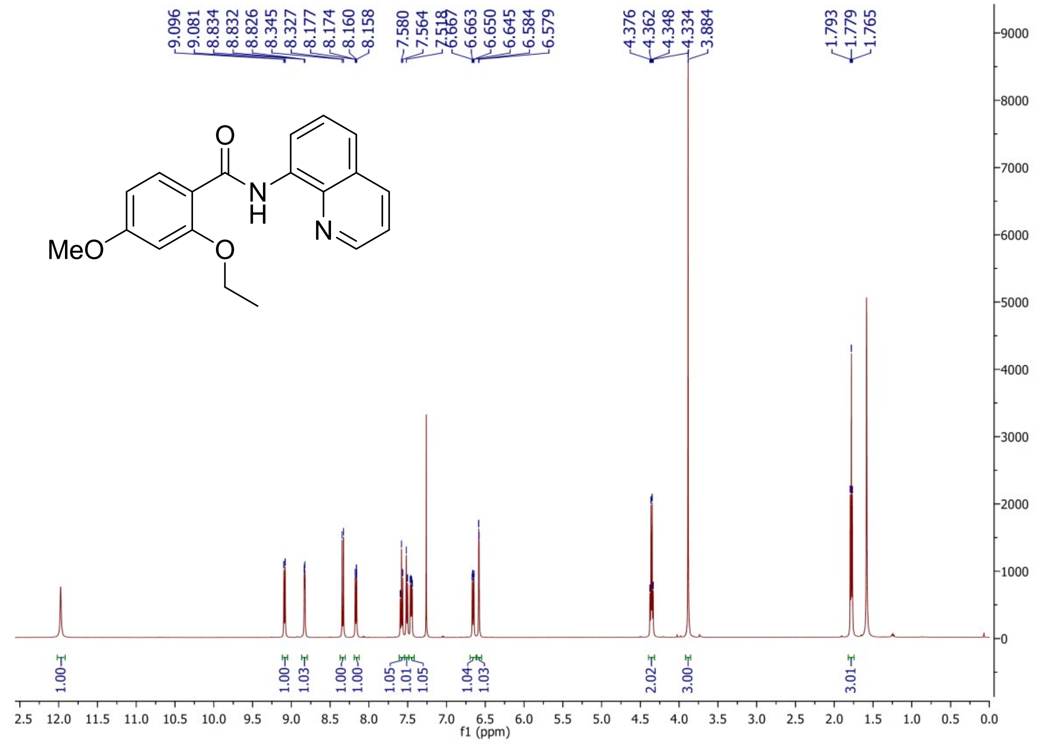

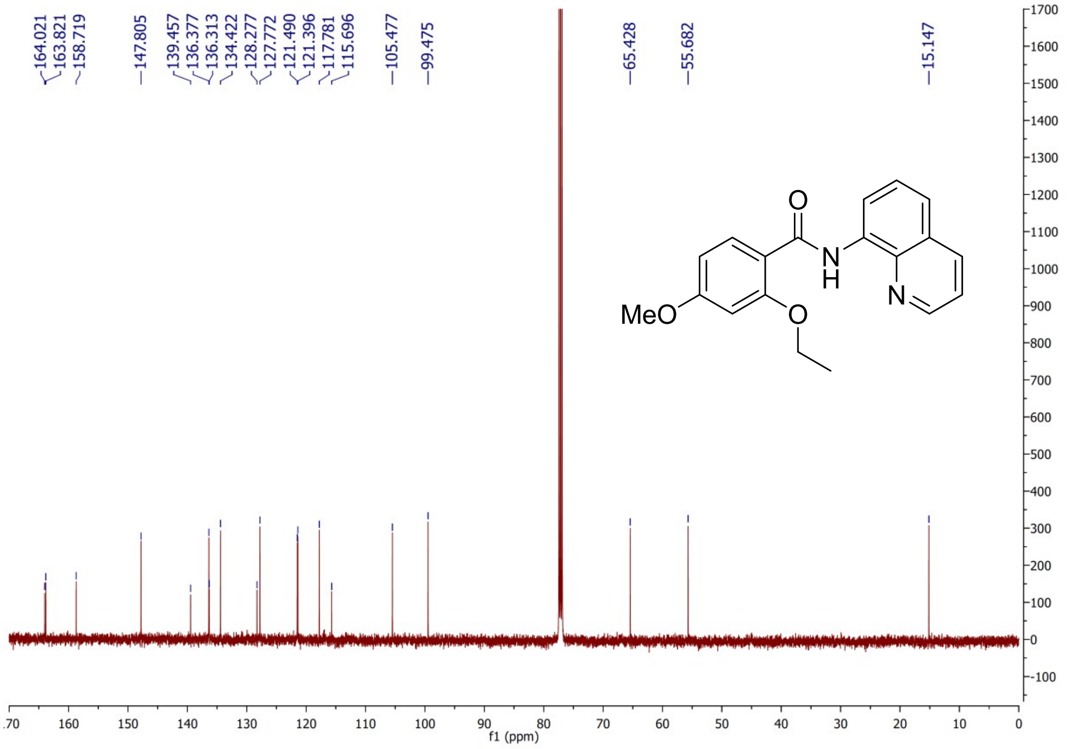

2-Methyl-*N*-(quinolin-8-yl)benzamide

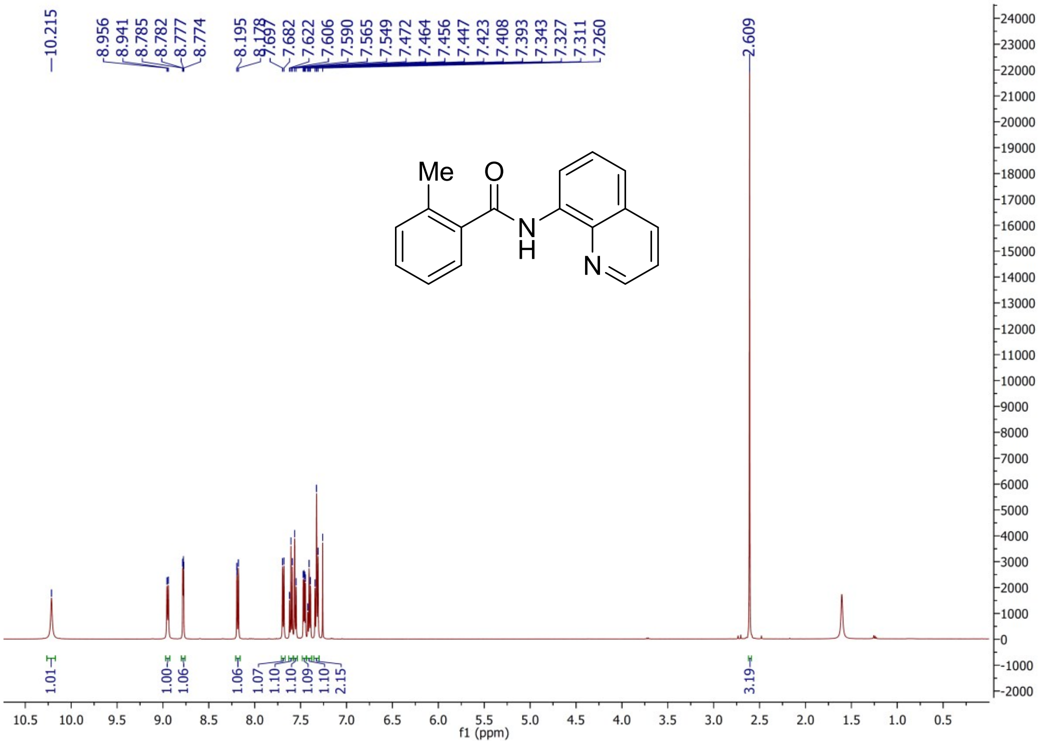

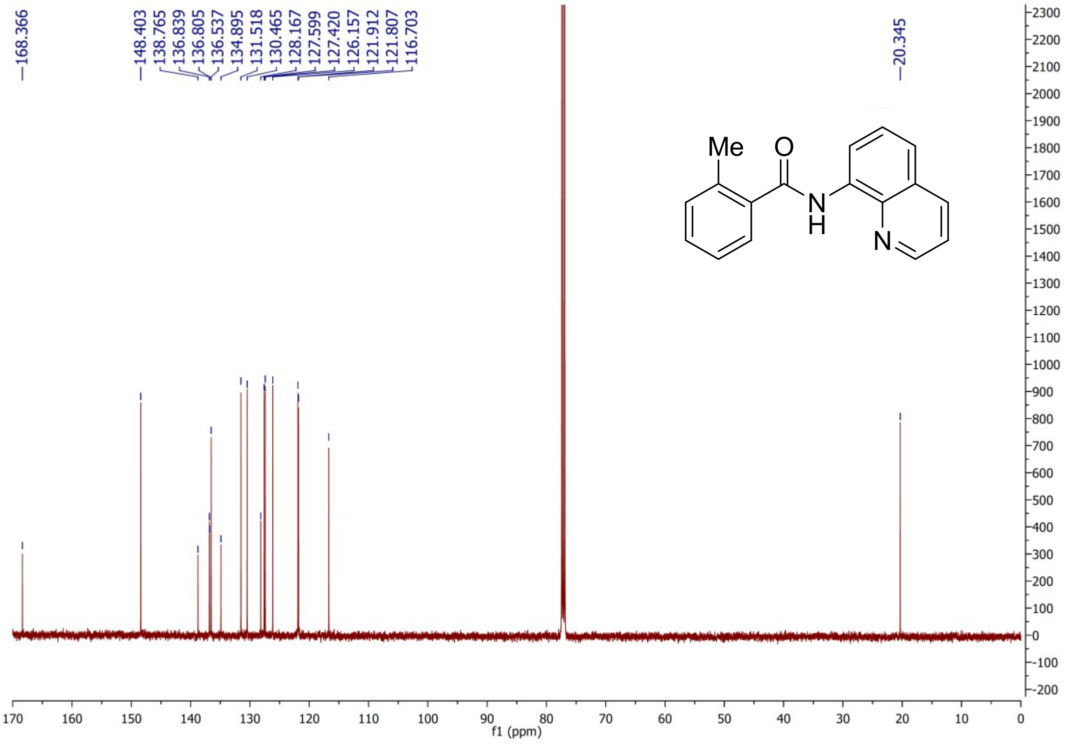

2-Ethoxy-4-methyl-*N*-(quinolin-8-yl)benzamide

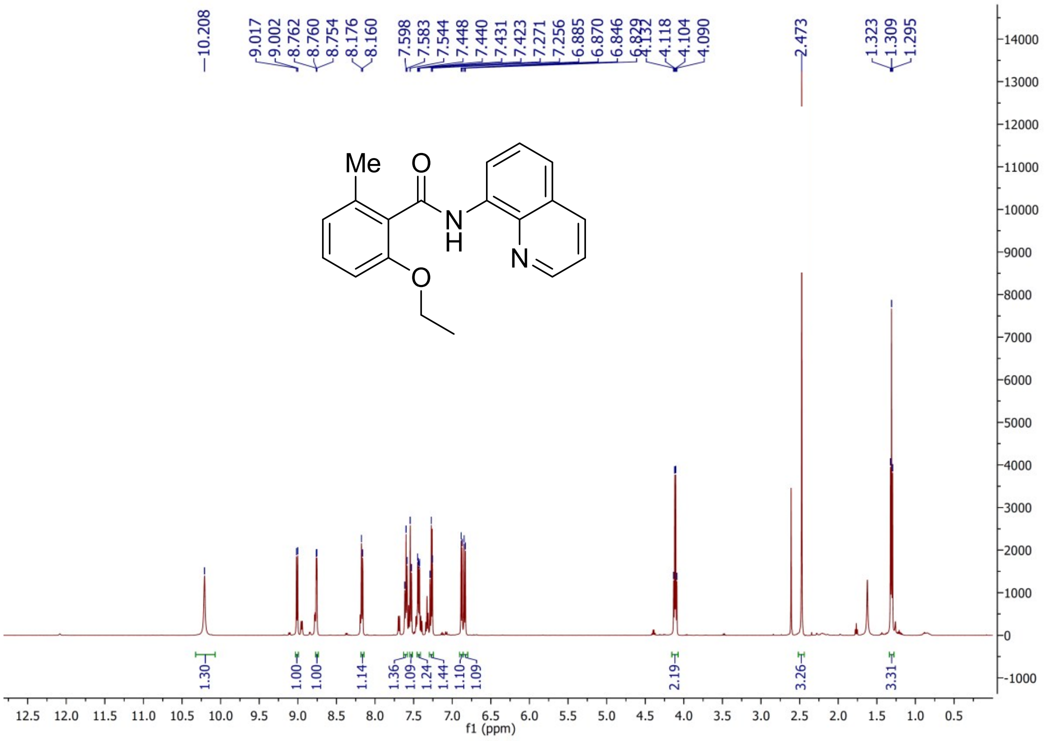

3-Bromo-*N*-(quinolin-8-yl)benzamide

3-Bromo-2-ethoxy-*N*-(quinolin-8-yl)benzamide

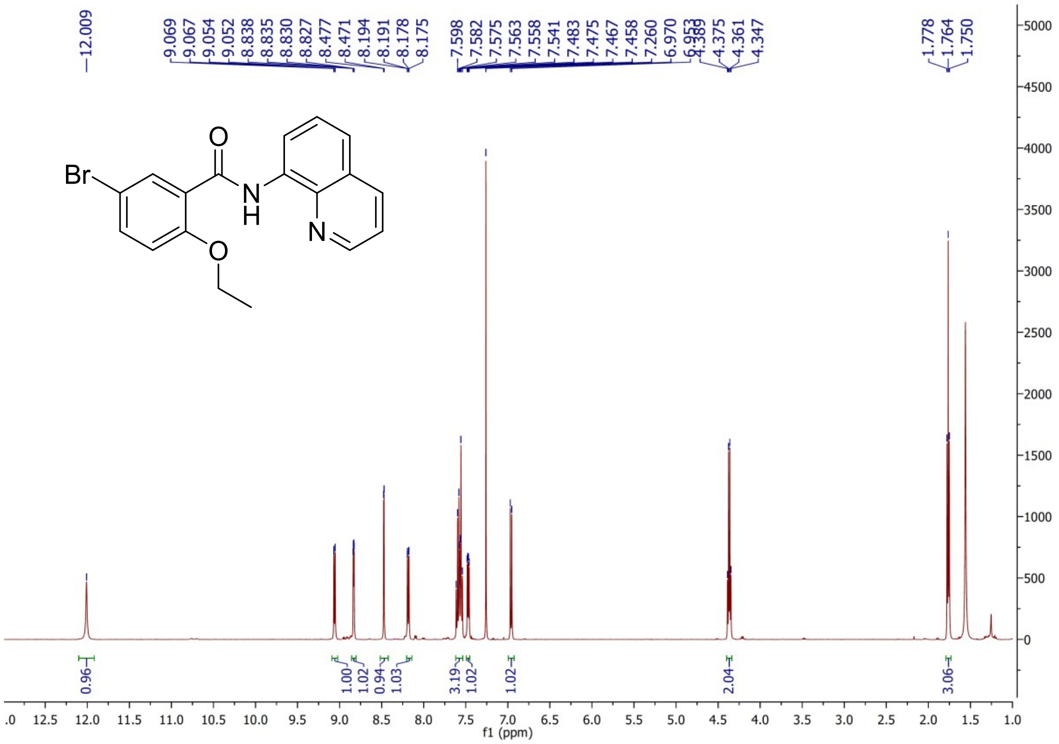

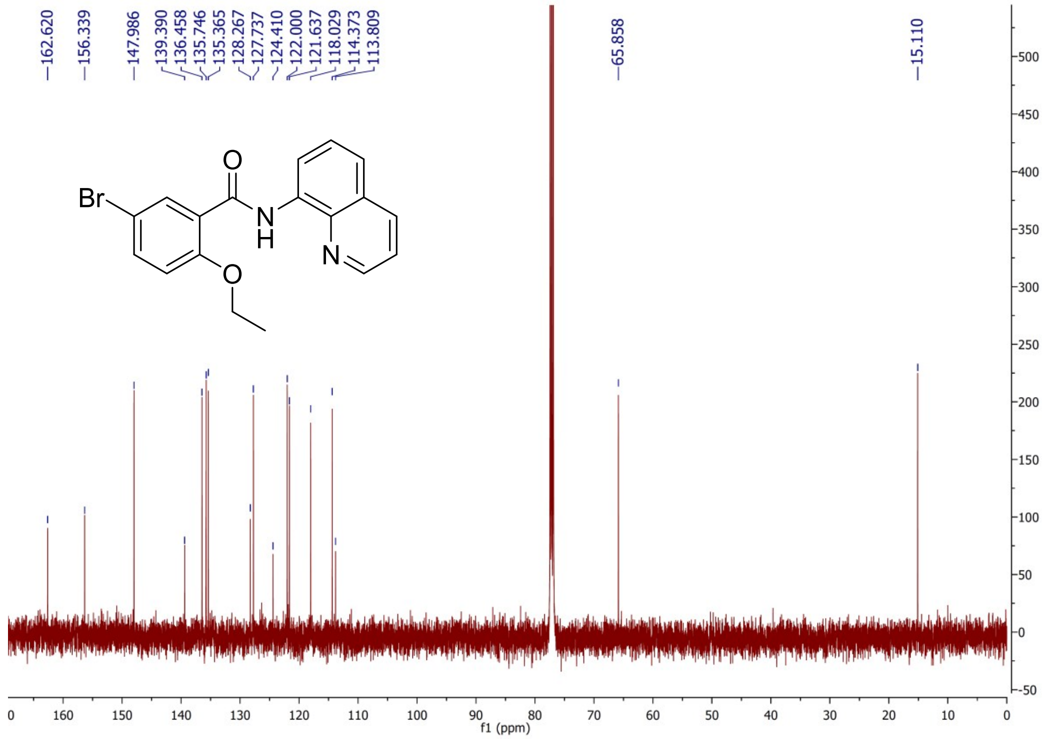

Supplement: RA-008-C7RA12010A-s001 [file RA-008-C7RA12010A-s001.pdf]
